# Design principles of improving the dose-response alignment in coupled GTPase switches

**DOI:** 10.1038/s41540-023-00266-9

**Published:** 2023-01-31

**Authors:** Lingxia Qiao, Pradipta Ghosh, Padmini Rangamani

**Affiliations:** 1 Department of Mechanical and Aerospace Engineering, Jacob’s School of Engineering, University of California San Diego, La Jolla, CA USA; 2 Department of Cellular and Molecular Medicine, School of Medicine, University of California San Diego, La Jolla, CA USA; 3 Moores Comprehensive Cancer Center, University of California San Diego, La Jolla, CA USA; 4 Department of Medicine, School of Medicine, University of California San Diego, La Jolla, CA USA

**Keywords:** Differential equations, Dynamical systems

## Abstract

“Dose-response alignment” (DoRA), where the downstream response of cellular signaling pathways closely matches the fraction of activated receptor, can improve the fidelity of dose information transmission. The negative feedback has been experimentally identified as a key component for DoRA, but numerical simulations indicate that negative feedback is not sufficient to achieve perfect DoRA, i.e., perfect match of downstream response and receptor activation level. Thus a natural question is whether there exist design principles for signaling motifs within only negative feedback loops to improve DoRA to near-perfect DoRA. Here, we investigated several model formulations of an experimentally validated circuit that couples two molecular switches—mGTPase (monomeric GTPase) and tGTPase (heterotrimeric GTPases) — with negative feedback loops. In the absence of feedback, the low and intermediate mGTPase activation levels benefit DoRA in mass action and Hill-function models, respectively. Adding negative feedback has versatile roles on DoRA: it may impair DoRA in the mass action model with low mGTPase activation level and Hill-function model with intermediate mGTPase activation level; in other cases, i.e., the mass action model with a high mGTPase activation level or the Hill-function model with a non-intermediate mGTPase activation level, it improves DoRA. Furthermore, we found that DoRA in a longer cascade (i.e., tGTPase) can be obtained using Hill-function kinetics under certain conditions. In summary, we show how ranges of activity of mGTPase, reaction kinetics, the negative feedback, and the cascade length affect DoRA. This work provides a framework for improving the DoRA performance in signaling motifs with negative feedback.

## Introduction

**Fig. 1:**
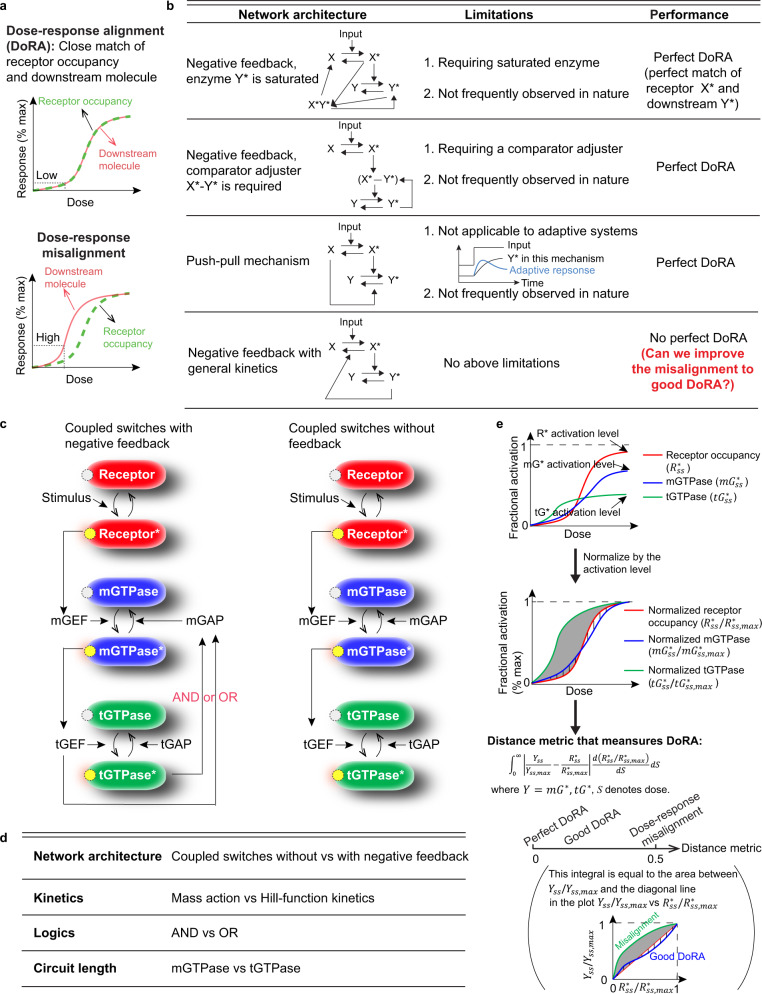
Exploring mechanisms for improving DoRA through GTPases switches. **a** The schematic of dose-response alignment (upper panel) and misalignment (lower panel). Dose-response alignment refers to the close match of receptor occupancy and the downstream molecules, and the misalignment the far distance between receptor occupancy and the downstream molecules. **b** Comparing network architectures capable of perfect DoRA to the negative feedback with general kinetics. “Perfect” means the perfect match of receptor occupancy and the downstream molecules. The naturally reoccurring negative feedback does not have so many constraints as the first three circuits, and we investigate how to improve misalignment to near-perfect DoRA in the circuit with negative feedback. **c** The experimentally identified coupled GTPase switches related to Arf1 (left) and the circuit without feedback (right). The adaptive response of Arf1 in^34^ is achieved by the negative feedback loops from active tGTPase to mGAP and from tGEF to mGAP. It should be noted that the cross-talk between two negative feedback loops can be modeled using logical AND or OR operations. **d** The kinetic details that may affect DoRA, depending on modeling choices that are explored in this work. **e** Distance metric that measures the DoRA performance: the upper panel shows dose-response curves of the fractional activation for receptors ${R}_{ss}^{* }$ (red) and downstream GTPases $m{G}_{ss}^{* }$ and $t{G}_{ss}^{* }$ (blue: mGTPase; green: tGTPase); these curves after normalization by their activation levels (defined as the level when the stimulus goes to infinity) are denoted by ${R}_{ss}^{* }/{R}_{ss,max}^{* }$ and *Y*_*ss*_/*Y*_*ss*,*max*_, *Y* = mG^*^, tG^*^, as shown in the middle panel; the lower panel defines the distance metric as the weighted sum of the distance between ${R}_{ss}^{* }/{R}_{ss,max}^{* }$ and *Y*_*ss*_/*Y*_*ss*,*max*_. This metric is equal to the area between the *Y*_*ss*_/*Y*_*ss*,*max*_ and the diagonal line in the plot *Y*_*ss*_/*Y*_*ss*,*max*_ vs ${R}_{ss}^{* }/{R}_{ss,max}^{* }$. The smaller value of this metric indicates better DoRA: zero means perfect DoRA, and small but non-zero value indicates a good DoRA.

One of the fundamental challenges in biology is understanding how cells reliably transmit chemical information from the extracellular milieu to the intracellular environment. The classical pathways begin with ligand-receptor interactions and involve complex biochemical reactions on the plasma membrane^1–5^. To capture the information transfer for these pathways, several metrics have been proposed. These metrics include dose response alignment (henceforth referred to as DoRA)^4,6–10^, the variance of downstream response^11–16^, and the channel capacity of signaling pathways^17–21^. Of these metrics, many of them focus on the stochastic behavior of downstream response while the assessment of the DoRA only requires the deterministic response for different levels of the stimulus. DoRA refers to the situation where the dose-response curves of the receptor occupancy and the downstream responses can be closely aligned (upper panel in Fig. 1a). In contrast, if these two curves are far away from each other, it is called “dose-response misalignment” (lower panel in Fig. 1a).

**Fig. 2:**
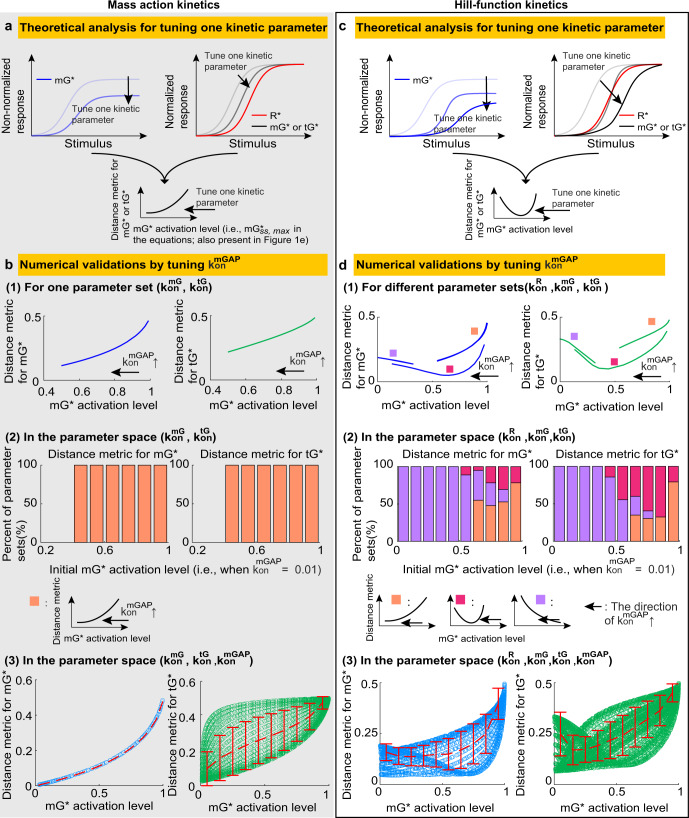
The relations between the DoRA behavior and the mGTPase activation level in the circuit without feedback. **a** The DoRA behavior improves with the decreased mGTPase activation level when tuning one kinetic parameter in the mass action model. **b** Numerical validations for results in A by tuning ${k}_{on}^{mGAP}$. The upper panel: the distance metric vs the mGTPase activation level when increasing ${k}_{on}^{mGAP}$ (denoted by ${k}_{on}^{mGAP}$*↑*) for a given parameter set. The middle panel: percents of maintaining the trend in the upper panel when searching the parameter space $\{{k}_{on}^{mG},{k}_{on}^{tG}\}$. The bottom panel: the distance metric vs the mGTPase activation level in the parameter space $\{{k}_{on}^{mG},{k}_{on}^{tG},{k}_{on}^{mGAP}\}$, with the dashed red line the mean and the error bar the standard deviation. See Supplementary Table 1 for parameters. **c** The DoRA behavior improves and then becomes bad with the decreased mGTPase activation level when tuning one kinetic parameter in the Hill-function model. **d** Same plot as in B except that the Hill-functions kinetics is adopted. The parameter space also expands to include the ${k}_{on}^{R}$, because the ${k}_{on}^{R}$ affects the receptor activation level and thus the mGTPase activation level in the Hill-function model.

DoRA has been found in many signaling systems, including the *Saccharomyces cerevisiae* pheromone pathway^4^, the insulin^22^, the thyrotropin^23^, angiotensin II^24^, and epidermal growth factor (EGF)^25,26^ response systems. The system exhibiting DoRA shows two advantages^4,6^: 1) the amplification of downstream response under the low dose is smaller than that in the absence of DoRA (dashed black lines in Fig. 1a); 2) the downstream response can sense the change of high doses, while the downstream response in the case of dose-response misalignment cannot due to the saturation under medium doses (Fig. 1a). Based on these observations, DoRA is believed to enhance the fidelity of information transmission^4^, indicating that DoRA might be a trait that is selected during the evolution of regulatory systems. Furthermore, the disruption of DoRA usually occurs when signaling pathways are perturbed: mutating the Wnt pathway by adding the inhibitor of GSK3*β* kinase interrupts the linear relation between *β*-catenin vs. phospho-LRP5/6 receptor, thus causing a far distance of the dose-response curves between *β*-catenin and phospho-LRP5/6 receptor^26^; the ERK pathway in the H1299 cell line expressing the mutated Raf-1 shows the nonlinear relation between EGF and ERK, which may lead to the misalignment of EGF receptor and ERK^26^. Although DoRA in other signaling pathways, such as the GTPase or NF*κ*B signaling pathways under EGF stimulus, remains to be experimentally validated, DoRA is desired to exist in signaling pathways where it facilitates fidelity of signal transduction.

Although the biochemical details of these systems are different, the idea that certain network topologies promote DoRA is appealing from the standpoint of identifying design principles. For example, studies have shown that the presence of a negative feedback loop is critical for DoRA^4,8,9^: the negative feedback loop can increase the level of stimulus that leads to half-maximal activation, which might be the reason for the achievement of DoRA. However, the negative feedback loop alone may not be able to achieve this alignment, and specific kinetics or comparator adjusters are required (first two circuits in Fig. 1b)^9^. A comparator adjuster can measure the difference between the downstream response and the receptor, and then adjust the downstream response to make it align with the receptor. The comparator adjuster has been built in a synthetic transcriptional cascade: the comparator utilizes the binding of anhydrotetracycline (ATc) and tetracycline repressor (TetR) to measure their concentration difference; the adjuster uses unbound TetR to inhibit the TetR synthesis to make total TetR align with the ATc^27^. Such an adjustment is analogous to “proportional control” in engineered systems, where the strength of negative feedback is adjusted to be proportional to the difference between output and input. Besides, a non-feedback topology—“push-pull” topology can also produce perfect DoRA (the third circuit in Fig. 1b), which was identified in^9^ and deeply dissected for coupled molecular switches in^7^. For this topology, the downstream response is not only upregulated (push) by the active receptors but also downregulated (pull) by the inactive receptors^7,9,28^. This mechanism has been validated in *Saccharomyces cerevisiae* yeast cell, where the protein RGS — GTPase activating protein (GAP) protein works as the “pull” by accelerating GTP hydrolysis and thereby, terminating tGTPase signaling^28^. However, the above mechanisms of perfect DoRA are difficult to achieve in several signal transduction pathways. The first two mechanisms in Fig. 1b require saturated enzyme or the existence of a comparator adjuster, and the third mechanism – push-pull mechanism does not apply to the system exhibiting adaptive response. Therefore, for the frequently observed negative feedback in nature^29–33^, where all the above mechanisms may not exist, how to improve the DoRA behavior to near-perfect remains unclear (last row in Fig. 1b).

Here, we studied how the DoRA can be improved in experimentally validated negative feedback loops. This circuit couples mGTPase (monomeric GTPase) and tGTPase (heterotrimeric GTPases) switches with negative feedback (left panel in Fig. 1c), where the monomeric GTPase Arf1 shows adaptive response^34^. The reason we chose this circuit is because it widely exists in eukaryotic cells and is important for the secretory pathway and cell proliferation^34^; the network architecture – negative feedback is a biologically recurring motif, and thus findings from a thorough interrogation of this circuit may be broadly applicable also to other molecular switches, including Rho, Rac, Rab, Ran, and other members of the Ras superfamily of GTPases. GTPase switches can switch between GTP-bound (active) state and GDP-bound (inactive) state, which are central to signal transduction pathways through which ligand or stimulus information from the extracellular space is transmitted to the intracellular space and leads to cellular decision making^35–39^. Besides, unlike the simplified model used in^7,9^, where only single molecular switches were modeled, we incorporated guanine exchange factors (GEFs) and GTPase activating proteins (GAPs), which catalyze the switch from the inactive to the active state and the reverse reaction, respectively. We investigated several model formulations of this coupled network, including the circuit without or with the negative feedback, the choice of model equations, and the logic for two species co-regulating the same target (right panel in Fig. 1c and Fig. 1d). Through theoretical analyses and numerical simulations, we identified several DoRA design principles including the role of feedback and the length of the cascade in improving DoRA.

## Model development

We briefly describe the biochemical circuit that we study here; this circuit was originally described in mammalian cells and experimentally interrogated in^40^, (left panel in Fig. 1c). In the presence of epidermal growth factor (EGF), the active Ras-superfamily mGTPases Arf1 on Golgi membrane recruit GIV/Girdin (a protein that is known to fuel aggressive traits in diverse cancers), and the latter works as guanine nucleotide exchange factor (GEF) to turn tGTPases Gi$\alpha \beta \gamma$ on^41–45^. Subsequently, GIV increases the level of the GAP for mGTPase by molecular scaffolding action, and the activated tGTPase acts as a co-factor to maximally activate the GAP. This circuit was subsequently modeled for cell secretion^34^ and for stability analysis^45^.

To translate this circuit into a mathematical model, we make the following assumptions. The total number of active and inactive receptors is assumed to be a constant, and so is the total number of mGTPases and that of tGTPases. Based on these assumptions, we choose the fractional activation, which is defined as the ratio of the number in the active form to the total number, to describe the state of the receptor or GTPase. The fraction of the inactive form is one minus the state variable.

### Governing equations

The dynamics of mGTPases, tGTPases, and corresponding GEFs and GAPs can be governed by the following system of equations.

1 $$\frac{d{R}^{* }}{dt}={k}_{on}^{R}f(S)[1-{R}^{* }]-{k}_{off}^{R}{R}^{* }$$

2 $$\frac{dmGEF}{dt}={k}_{on}^{mGEF}f({R}^{* })-{k}_{off}^{mGEF}mGEF$$

3 $$\frac{dm{G}^{* }}{dt}={k}_{on}^{mG}f(mGEF)[1-m{G}^{* }]-{k}_{off}^{mG}f(mGAP)m{G}^{* }$$

4 $$\frac{dtGEF}{dt}={k}_{on}^{tGEF}f(m{G}^{* })-{k}_{off}^{tGEF}tGEF$$

5 $$\frac{dt{G}^{* }}{dt}={k}_{on}^{tG}f(tGEF)[1-t{G}^{* }]-{k}_{off}^{tG}f(tGAP)t{G}^{* }$$

6 $$\frac{dmGAP}{dt}={k}_{on}^{mGAP}+{k}_{feedback}F(tGEF,t{G}^{* })-{k}_{off}^{mGAP}mGAP$$

where *S* denotes the stimulus EGF. R^*^, mG^*^, tG^*^ represent the fractional activation of the receptor, mGTPase, and tGTPase, respectively. *mGEF*, *tGEF*, *mGAP* are concentrations of GEF for mGTPase (mGEF), GEF for tGTPase (i.e., GIV; denoted as tGEF), GAP for mGTPase (mGAP), respectively. *k*_*on*_’s are production rate constants, *k*_*off*_’s are decay rate constants. The negative feedback loops from the active tGTPase and tGEF to mGAP are modeled by *k*_*feedback*_*F*(*tGEF*, tG^*^), where *k*_*feedback*_ indicates the negative feedback strength and *F*(*tGEF*, tG^*^) the crosstalk between tGEF and active tGTPase. The function *f* describes the effect of the regulator.

#### Form of reaction kinetics f

The outcome of a model for signal transduction depends on the form of the reaction kinetics. The activation or deactivation of GTPase is a linear function with GEF or GAP when GEF or GAP is in certain ranges^46^, and many studies built the model based on mass action kinetics^7,26^. However, it can be nonlinear if GEF or GAP varies over large ranges^47,48^, where mass action kinetics is not suitable. Therefore, it is hard to determine which kinetics these reactions are without further and detailed experiments. Since both mass action and Hill-function kinetics have been widely used in modeling reaction rates^7,9,49,50^, here, we explore the coupled GTPase circuit using two classic forms of reaction kinetics – mass action and Hill functions. When the model is developed using mass action kinetics *f* in Eq. (1)-Eq. (6), the *f* becomes

$$f(x)=x.$$

For the Hill-function kinetics,

$$f(x)\,\triangleq\, {f}_{act}(x)=\frac{{x}^{n}}{{K}^{n}+{x}^{n}}$$

Here *K* and *n* are half-maximal activation and Hill coefficient, respectively.

#### AND and OR logic gates to model feedback term *F*(*tGEF*, tG^*^)

While it is known that tGEF and active tGTPase are both required to exert mGAP^40^, the advantage of such AND logic between *tGEF* → *mGAP* and tG^*^ → *mGAP* is not yet clear. Therefore, we consider two options for the feedback by studying the AND logic gate and the OR logic gate. If the AND logic gate is used to model this interaction, *f*(*tGEF*) and *f*(tG^*^) are multiplied together; if the OR logic gate is applied, these two terms are added together. As a result, we can write the *F*(*tGEF*, tG^*^) as follows:

$$F(tGEF,t{G}^{* })=\left\{\begin{array}{ll}f(tGEF)f(t{G}^{* })\quad &\,{{\mbox{for the AND logic gate}}}\,\\ f(tGEF)+f(t{G}^{* })\quad &\,{{\mbox{for the OR logic gate}}}\,\end{array}\right.$$

To investigate the role of the negative feedback loops on the DoRA, we also perturbed this circuit by deleting the negative feedback loops (right panel Fig. 1c), and this is achieved by setting *F*(*tGEF*, tG^*^) ≡ 0.

Thus, we have six models according to different combinations of *f* and *F*(*tGEF*, tG^*^), because *f* and *F*(*tGEF*, tG^*^) have two and three choices, respectively. The three choices of *F*(*tGEF*, tG^*^), i.e., 0, *f*(*tGEF*)*f*(tG^*^) or *f*(*tGEF*) + *f*(tG^*^), correspond to the circuit without feedback, the circuit whose negative feedback is modeled by AND logic gate, and the circuit where the OR logic gate is applied. For each circuit, *f* has two choices, indicating which kinetics is adopted. See section “Methods” for the numerical simulations for these models.

### Distance metric to measure DoRA performance

The dose-response alignment for species downstream of the receptor can be obtained from inspecting how well dose-response curve of the downstream species is aligned with (or closely matches) the receptor occupancy curve. To quantitatively measure the DoRA performance, we defined the following “distance metric”, which is the weighted sum of the absolute value between the “normalized” GTPase and the “normalized” receptor dose-response curves (Fig. 1e):

$$\,{{\mbox{Distance metric}}}\,={\int}_{S}\left\vert \frac{{Y}_{ss}}{{Y}_{ss,max}}-\frac{{R}_{ss}^{* }}{{R}_{ss,max}^{* }}\right\vert \frac{d{R}_{ss}^{* }/{R}_{ss,max}^{* }}{dS}dS.$$

where *Y* can be mG^*^, tG^*^. The subscript *ss* denotes the steady-state value of the fractional activation, and the subscript *max* the maximal steady-state value of fractional activation for receptors and GTPases for all doses *S*. For simplicity, the $m{G}_{ss,max}^{* }$ is referred to as the mGTPase activation level. The smaller this metric is, the better the DoRA performance is. Therefore, the zero value of the distance metric indicates the perfect match of normalized dose-response curves between GTPase and the receptor, i.e., the perfect DoRA; a small but non-zero value means a good DoRA performance. If the variable of integration *S* is replaced by ${R}_{ss}^{* }/{R}_{ss,max}^{* }$, it can be seen that this definition is equal to the area between the diagonal line and the curve of the normalized GTPase response versus the normalized receptor response (the panel in the bracket in Fig. 1e). Besides, this definition is the integral of the absolute deviations in the vertical direction, while the SWRMS distance metric defined by Andrews et al.^9^ is the integral of the squared deviations in both vertical and horizontal directions. One advantage of the metric used in our work is that it makes detailed theoretical analyses feasible due to its simple form.

## Results

### mGTPase activation levels impact DoRA in the absence of negative feedback

We begin our analysis with the simple case of coupled switches without any negative feedback (right panel in Fig. 1c) for both mass action and Hill-type kinetics. Yu et al. showed that when the downstream response has a small value, the distance of normalized dose-response curves between the receptor and downstream response is small, leading to a good DoRA performance^4^. Therefore, we first investigated how GTPase activation levels determine GTPases’ DoRA behavior. Here, we only considered the mGTPase activation level, because the positive regulation from mGTPase to tGTPase leads to a positive correlation between m- and tGTPase activation levels.

First, in the mass action model, the DoRA can be improved by decreasing the mGTPase level. The normalized steady-state values of mG^*^ and tG^*^ are given by (see “Methods” for details)

7 $$\frac{m{G}_{ss}^{* }}{m{G}_{ss,max}^{* }}=\frac{{R}_{ss}^{* }}{1-m{G}_{ss,max}^{* }(1-{R}_{ss}^{* })},\quad \frac{t{G}_{ss}^{* }}{t{G}_{ss,max}^{* }}={R}_{ss}^{* }\frac{1+\left(\frac{1}{m{G}_{ss,max}^{* }}-1\right)\frac{{b}_{M}}{1+{b}_{M}}}{{R}_{ss}^{* }+\left(\frac{1}{m{G}_{ss,max}^{* }}-1\right)\frac{{b}_{M}}{1+{b}_{M}}}.$$

where the subscripts *ss* and *max* denote the steady-state value and the maximal value, respectively. ${b}_{M}=tGAP\frac{{k}_{off}^{tGEF}{k}_{off}^{tG}}{{k}_{on}^{tG}{k}_{on}^{tGEF}}$, which is independent with the mGTPase activation level $m{G}_{ss,max}^{* }$ (see also “Distance metric to measure DoRA performance” section for the definition of $m{G}_{ss,max}^{* }$). According to these two equations, the normalized m- and tGTPases, i.e., $\frac{m{G}_{ss}^{* }}{m{G}_{ss,max}^{* }}$ and $\frac{t{G}_{ss}^{* }}{t{G}_{ss,max}^{* }}$, both have two important properties (Fig. 2a): the first is that they are always larger than ${R}_{ss}^{* }/{R}_{ss,max}^{* }$ (${R}_{ss,max}^{* }$ is 1 in the mass action model), and the second is that their values decrease with decreased mGTPase activation level $m{G}_{ss,max}^{* }$. These two properties suggest that the small mGTPase activation level lowers the normalized m- and tGTPase curves and thus makes them close to the receptor curve, resulting in good DoRA behavior for both GTPases. Furthermore, if we tune one kinetic parameter to lower the mGTPase activation level, the DoRA for both GTPases can be enhanced (Fig. 2a).

We further validated the above analysis numerically by studying the effects of only tuning ${k}_{on}^{mGAP}$. For a given parameter set, increasing ${k}_{on}^{mGAP}$ leads to not only the decrease in mGTPase activation level but also the improved DoRA behavior for both GTPases (the upper panel in Fig. 2b; also see Supplementary Fig. 1a for dose-response curves). This trend is not affected by the choice of parameter sets: for every $\{{k}_{on}^{mG},{k}_{on}^{tG}\}$ set, which may have different mGTPase activation levels when the ${k}_{on}^{mGAP}$ is 0.01, increasing ${k}_{on}^{mGAP}$ from 0.01 to 1 causes the decrease of both the distance metric and the mGTPase activation level (the middle panel in Fig. 2b). The above results indicate that when other kinetic parameters are fixed, increasing ${k}_{on}^{mGAP}$, i.e., decreasing mGTPase activation level, can enhance DoRA for both GTPases. This conclusion is not influenced by choosing which kinetic parameter to tune (Supplementary Fig. 2). Furthermore, to study the effects of tuning more than two kinetic parameters simultaneously instead of only tuning ${k}_{on}^{mGAP}$, we plotted the scatter plots of the mGTPase activation level vs the distance metric in the parameter space $\{{k}_{on}^{mG},{k}_{on}^{tG},{k}_{on}^{mGAP}\}$ (the bottom panel in Fig. 2b). The DoRA behavior of mGTPase can be improved as long as the mGTPase activation level is decreased, because there exists a one-to-one mapping from the mGTPase activation level to the mGTPase’s distance metric. Nevertheless, decreasing the mGTPase activation level can impair or improve the tGTPase’s DoRA behavior due to the one-to-many mapping, but the improvement effect has a larger probability to occur according to the mean trend of the distance metric.

Next, we turned to the Hill-function model and found that an intermediate mGTPase activation level benefits the DoRA for the circuit without feedback. In the Hill-function model, the mGTPase activation level $m{G}_{ss,max}^{* }$ and the $\frac{m{G}_{ss}^{* }}{m{G}_{ss,max}^{* }}$ are given by (see “Methods” for details):

8 $$m{G}_{ss,max}^{* }=\frac{{F}_{1}({R}_{ss,max}^{* })}{{F}_{1}({R}_{ss,max}^{* })+{a}_{H}},\quad \frac{m{G}_{ss}^{* }}{m{G}_{ss,max}^{* }}=\frac{{F}_{1}({R}_{ss}^{* })}{{F}_{1}({R}_{ss,max}^{* })}\frac{{F}_{1}({R}_{ss,max}^{* })+{a}_{H}}{{F}_{1}({R}_{ss}^{* })+{a}_{H}},$$

where ${F}_{1}({R}_{ss}^{* })={f}_{act}(\frac{{k}_{on}^{mGEF}}{{k}_{off}^{mGEF}}{f}_{act}({R}_{ss}^{* }))$, ${a}_{H}=\frac{{k}_{on}^{mG}}{{k}_{off}^{mG}}{f}_{act}(\frac{{k}_{on}^{mGAP}}{{k}_{off}^{mGAP}})$, and ${b}_{H}=\frac{{k}_{on}^{tG}}{{k}_{off}^{tG}}{f}_{act}(tGAP)$. Unlike the case for the mass action model where we can rewrite $\frac{m{G}_{ss}^{* }}{m{G}_{ss,max}^{* }}$ as a function of $m{G}_{ss,max}^{* }$, in the Hill-function model, we can only vary one kinetic parameter and study the corresponding changes of $m{G}_{ss,max}^{* }$ and $\frac{m{G}_{ss}^{* }}{m{G}_{ss,max}^{* }}$. By deriving derivatives of $\frac{m{G}_{ss}^{* }}{m{G}_{ss,max}^{* }}$ with respect to each kinetic parameter (Eq. (20)), we proved that $\frac{m{G}_{ss}^{* }}{m{G}_{ss,max}^{* }}$ will decrease when one kinetic parameter is tuned to reduce the $m{G}_{ss,max}^{* }$ (Fig. 2c). However, due to the nonlinearity of the *F*_1_, the normalized GTPase curves may cross the receptor curve from the left to the right when decreasing $m{G}_{ss,max}^{* }$, suggesting that the DoRA behavior improves and then becomes worse as the mGTPase activation level $m{G}_{ss,max}^{* }$ decreases (Fig. 2c). Therefore, an intermediate mGTPase activation level results in good DoRA performance for the Hill-function modeled circuit in the absence of feedback.

Similar to the numerical validations in the mass action model, we also took ${k}_{on}^{mGAP}$ as an example to verify the above analysis for the Hill-function model. For three different parameter sets of $\{{k}_{on}^{R},{k}_{on}^{mG},{k}_{on}^{tG}\}$, increasing the ${k}_{on}^{mGAP}$ from 0.01 to 1 all reduces the mGTPase activation level, but the distance metric for both GTPases can be decreasing, increasing, or decreasing at first and then increasing (the upper panel in Fig. 2d; also see Supplementary Fig. 1b). Note that the decreasing trend of the distance metric with increased ${k}_{on}^{mGAP}$ tends to have large mGTPase activation levels, the increasing trend the small mGTPase activation levels, and the non-monotonic trend the intermediate mGTPase activation levels. This also holds when we sampled more parameter sets of $\{{k}_{on}^{R},{k}_{on}^{mG},{k}_{on}^{tG}\}$ in the whole parameter space (the middle panel in Fig. 2d): the decreasing, increasing, and non-monotonic trends are located in high, low, and intermediate initial mGTPase activation level (the value when ${k}_{on}^{mGAP}=0.01$), respectively. These simulations indicate that an intermediate mGTPase activation is preferred for the DoRA behavior, which also holds when tuning other kinetic parameter rather than ${k}_{on}^{mGAP}$ (Supplementary Fig. 3). Furthermore, in the parameter space $\{{k}_{on}^{R},{k}_{on}^{mG},{k}_{on}^{tG},{k}_{on}^{mGAP}\}$, the relation between the mean DoRA behavior and the mGTPase activation level shows consistent results (the bottom panel in Fig. 2d).

### The effect of negative feedback is model-dependent and mGTPase activation level-dependent

The above analyses focused on the coupled switches without feedback. Next, we will investigate the effect of adding negative feedback. Although the effects of the feedback is the same as the high ${k}_{on}^{mGAP}$ value for the circuit without feedback based on their inhibitory roles in the mGTPase activation level, their effects on the DoRA may differ a lot because the feedback induces more non-linearity. The strength of the feedback is tuned by varying *k*_*feedback*_, where 0 means no feedback and a large value indicates strong feedback. Moreover, as we have shown in the previous section, different kinetics forms lead to different DoRA performance, so the mass action and Hill-function kinetics are both considered.

In the mass action model, adding negative feedback has diverse effects on DoRA: the negative feedback enhances (or impairs) DoRA behavior when the mGTPase activation level is high (or low), while the intermediate mGTPase activation level leads to the non-monotonic effect of negative feedback. The theoretical analyses are summarized as follows (see “Methods” for details). The existence of feedback prevents us from directly obtaining how the feedback strength *k*_*feedback*_ affects $\frac{m{G}_{ss}^{* }}{m{G}_{ss,max}^{* }}$, so we calculated $\frac{\partial }{\partial {k}_{feedback}}\frac{m{G}_{ss}^{* }}{m{G}_{ss,max}^{* }}$ and decomposed it into two parts:

$$\frac{\partial }{\partial {k}_{feedback}}\frac{m{G}_{ss}^{* }}{m{G}_{ss,max}^{* }}=\left(\frac{1}{m{G}_{ss}^{* }}\frac{\partial m{G}_{ss}^{* }}{\partial {k}_{feedback}}-\frac{1}{m{G}_{ss,max}^{* }}\frac{\partial m{G}_{ss,max}^{* }}{\partial {k}_{feedback}}\right)\frac{m{G}_{ss}^{* }}{m{G}_{ss,max}^{* }}.$$

**Fig. 3:**
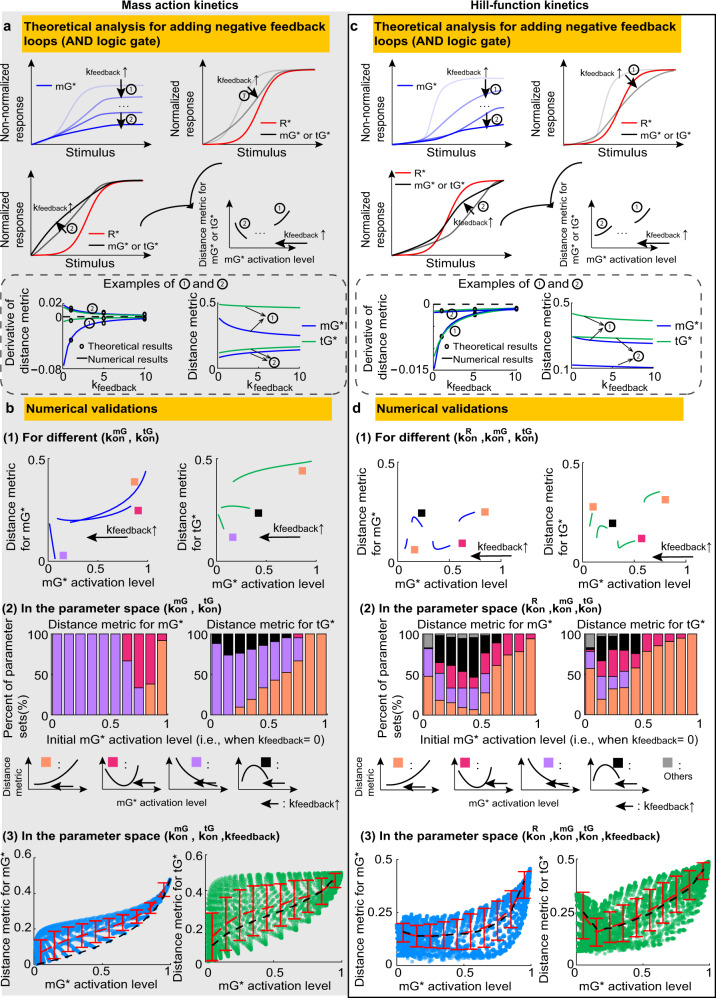
Effects of adding the negative feedback. Same plot as in Fig. 2, except that the feedback strength *k*_*feedback*_ is tuned while maintaining other kinetic parameters unchanged. The three dots in (**a**) indicate the region where the nonmonotonic change of the distance metric may occur. The bottom panels in (**a**) and (**c**) show how the derivative of the distance metric and the distance metric itself are determined by *k*_*feedback*_ under different parameters; the derivative obtained from the formula is the same as that from the finite difference. In panels **b**(3) and **d**(3), the number of kinetic parameter sets is one-fourth of that in Fig. 2b(3) and Fig. 2d(3), and black dashed lines are the red dashed lines in Fig. 2b(3) and Fig. 2d(3). Here, the AND logic gate is used to model the negative feedback loops. See Supplementary Table 1 for parameters.

The term in the bracket is the difference of the function $\frac{1}{m{G}_{ss}^{* }}\frac{\partial m{G}_{ss}^{* }}{\partial {k}_{feedback}}$ at $m{G}_{ss}^{* }$ and $m{G}_{ss,max}^{* }$. Since this function decreases at first and then increases with increased $m{G}_{ss}^{* }$ (Supplementary Figs. 4–7, and Eq. (21)), the sign of $\frac{\partial }{\partial {k}_{feedback}}\frac{m{G}_{ss}^{* }}{m{G}_{ss,max}^{* }}$ is positive for small $m{G}_{ss}^{* }$ and negative for large $m{G}_{ss}^{* }$. Therefore, if the mGTPase activation level $m{G}_{ss,max}^{* }$ is high enough, the normalized mGTPase curve $\frac{m{G}_{ss}^{* }}{m{G}_{ss,max}^{* }}$ shows the decreasing trend with increased feedback strength, indicating the positive role of feedback in the DoRA performance for mGTPase (① in Fig. 3a; also see Supplementary Fig. 4). Similarly, when the mGTPase activation level is low, the feedback exhibits negative effect on the DoRA performance for mGTPase (② in Fig. 3a; also see Supplementary Fig. 5). When the mGTPase activation level is neither high nor low, nonmonotonic effect occurs. Similar conclusions can be drawn for tGTPase (see “Methods”).

We also validated above conclusions through numerical simulations. For different values of ${k}_{on}^{mG}$ and ${k}_{on}^{tG}$, increasing feedback strength (i.e., increasing *k*_*feedbak*_ from 0 to 10^2.6^) all causes the decrease of the mGTPase activation level, but trends for the distance metric are diverse (upper and middle panels in Fig. 3b; also see Supplementary Fig. 1c): increasing (purple), decreasing (orange), decreasing first and then increasing (magenta), or increasing first and then decreasing (black). Furthermore, increasing and decreasing trends usually occur when the mGTPase activation level in the absence of feedback is low and high, respectively. These results are consistent with the theoretical analyses. Though we elaborated on AND logic gate, the results are the same when using OR logic gate (Supplementary Fig. 8a, b).

The above analyses focus on the trend of DoRA performance when only tuning feedback strength and keeping other parameters unchanged. Next, we investigated the role of feedback in the larger parameter space. We found that the mean distance metric for the circuit with negative feedback (red dashed line in the bottom panel in Fig. 3b) is higher than that without feedback (the black dashed line in the bottom panel in Fig. 3b). This indicates that if the mGTPase levels are the same, the circuit with negative feedback loops exhibits worse mean DoRA performance than that without feedback. It should be noted that the kinetic parameters with the same mGTPase levels can be totally different from each other. In other words, if the kinetic parameters are randomly assigned to ensure the same mGTPase activation level, the circuit with negative feedback shows worse DoRA performance than the circuit without feedback. On the contrary, as shown in the upper and middle panels in Fig. 3b, if the kinetic parameters are fixed for the circuit without feedback, adding negative feedback loops may enhance or impair DoRA performance but with the expense of the mGTPase activation level.

Next, we turned to the Hill-function model to explore the role of negative feedback. Unlike the case in the mass action model, adding negative feedback in the Hill-function model usually enhances the DoRA behavior when the mGTPase activation level is high or low. In the Hill-function model, the $\frac{\partial }{\partial {k}_{feedback}}\frac{m{G}_{ss}^{* }}{m{G}_{ss,max}^{* }}$ follows the same proprieties as in the mass action model (see “Methods”), but $\frac{m{G}_{ss}^{* }}{m{G}_{ss,max}^{* }}$ can across the receptor curve $\frac{{R}_{ss}^{* }}{{R}_{ss,max}^{* }}$ depending on the mGTPase activation level $m{G}_{ss,max}^{* }$. Taken together, the high mGTPase activation level usually corresponds to a higher location of the mGTPase curve than the receptor curve, and increasing *k*_*feedback*_ under this condition decreases the location of the mGTPase curve $\frac{m{G}_{ss}^{* }}{m{G}_{ss,max}^{* }}$, leading to a good DoRA performance of mGTPase (the ① in Fig. 3c). On the other hand, the low mGTPase activation level often means a lower location of the mGTPase curve than the receptor curve, and increasing *k*_*feedback*_ at this time raises the mGTPase curve (see “Methods”), also ensuring the positive role of negative feedback for the DoRA performance of mGTPase (the ② in Fig. 3c). Similar conclusions can be drawn for tGTPase (see “Methods”).

Numerical simulations support the above conclusions. Here, we demonstrated the AND logic gate to model the negative feedback loops, while the OR logic gate shows similar results (Supplementary Fig. 8c, e). For two given parameter sets with either high or low mGTPase activation level (marked by orange boxes in the upper panel in Fig. 3d; Supplementary Fig. 1d), increasing the feedback strength, i.e., varying *k*_*feedback*_ from 0 to 10^2.6^, decreases the distance metric; in the whole parameter space $\{{k}_{on}^{R},{k}_{on}^{mG},{k}_{on}^{tG}\}$, the high or extremely low mGTPase activation level often leads to the decreasing trend of the distance metric (orange bars in the middle panel in Fig. 3d). Since the mass action kinetics shows such decreasing trend of the distance metric only when the mGTPase activation level is high, these results suggest that the Hill-function kinetics has a larger parameter space to produce this decreasing trend. Therefore, compared with the mass action model, the effect of negative feedback in the Hill-function model is more unified - improving DoRA performance in most cases. However, when the mGTPase activation level is in the intermediate range, trends of the distance metric with respect to the feedback strength are non-monotonic and diverse (magenta, black and gray colors in Fig. 3d; Supplementary Fig. 1d).

Furthermore, we compared the mean DoRA behavior for the circuit without or with negative feedback in the whole parameter space, where both circuits are still modeled by Hill-function kinetics. The mean DoRA metrics for the circuit without or with negative feedback are almost overlapped in the Hill-function model (in the lower panel in Fig. 3d), while in the mass action model the mean DoRA metrics for these two circuits are far from each other. This indicates that Hill-function kinetics leads to a smaller effect of negative feedback on the mean DoRA performance compared with the mass action kinetics. This may result from the saturation effect of the Hill function; the whole system cannot respond to further changes in the feedback strength when the system is saturated.

### The OR logic gate has the similar DoRA behavior to the AND logic gate

**Fig. 4:**
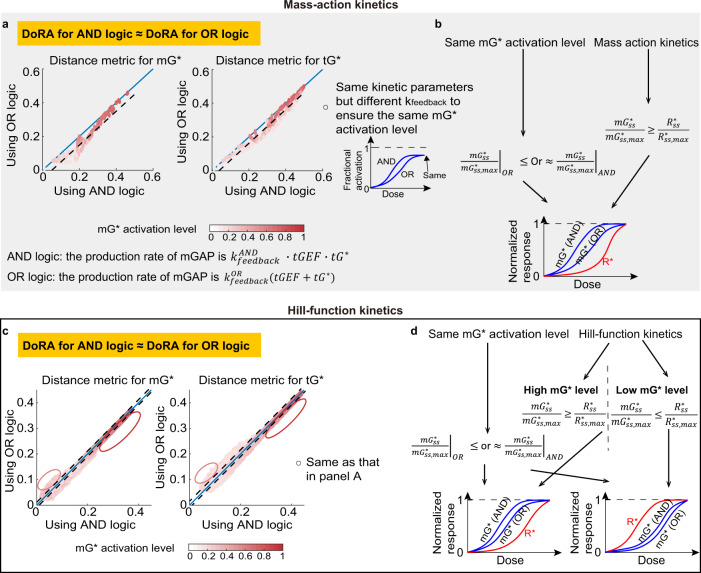
The OR logic gate has the similar DoRA behavior to the AND logic gate. **a** Comparisons of the distance metrics between the system with the AND logic gate and that with the OR logic gate. The AND logic gate means that the production rate of mGAP is modeled by ${k}_{feedback}^{AND}\cdot tGEF\cdot t{G}^{* }$; the OR logic gate corresponds to ${k}_{feedback}^{OR}(tGEF+t{G}^{* })$. Each dot corresponds to one kinetic parameter set in the parameter space $\{{k}_{on}^{R},{k}_{on}^{mG},{k}_{on}^{tG}\}$, and the ${k}_{feedback}^{OR}$ is different from ${k}_{feedback}^{AND}$ to ensure the same mGTPase activation level $m{G}_{ss,max}^{* }$ (the value when the stimulus *S* → *∞*). The black dashed line indicates the averaged distance from dots to the diagonal line. The diagonal line is in blue. **b** Theoretical analysis for comparisons of distance metrics between AND and OR logic gates in the mass action model. The *tGEF* can be rewritten as a function of mG^*^, i.e., *h*_1_(mG^*^); the tG^*^ is rewritten as the function *h*_2_(mG^*^). **c**, **d** The same plots as in (**a**–**b**) but the Hill-function kinetics is adopted.

While modeling the negative feedback loops with the AND or OR logic gate does not change the trend of the DoRA behavior with increased feedback strength, the DoRA behavior with a certain feedback strength may differ. To make a fair comparison, for the system with the AND or OR logic gate, we chose the same parameter set but allowed different values of *k*_*feedback*_ to ensure the same mGTPase activation level $m{G}_{ss,max}^{* }$. This constraint is based on the importance of the mGTPase activation level as shown in previous sections. Once we have such kinetic parameters and the different *k*_*feedback*_, we computed and compared the distance metrics between these two systems (corresponding to one dot in Fig. 4a). After we randomly chose kinetic parameter sets and corresponding *k*_*feedback*_, we can compare several pairs of distance metrics for the systems using different logic gates (Fig. 4a).

Interestingly, in the mass action model, the AND and OR logic gates show almost the same DoRA behavior. This is demonstrated by the small distance between the diagonal line and dots in Fig. 4a, where x and y coordinates of these dots are the distance metric for the AND and OR logic gates, respectively. Next, we validated this finding theoretically. Though the OR logic gate shows better DoRA performance than AND logic gate (see “Methods” and Eq. (22)), this advantage of OR logic gate is negligible if the feedback strength is close to zero (the dots in dark red in Fig. 4a), because the model using the OR or AND logic gate both degenerates to the same model in the absence of the feedback. When the feedback strength is nonzero, it seems that the DoRA performance for the AND logic gate and that for the OR logic gate are still almost the same. This might come from the almost same $m{G}_{ss}^{* }/m{G}_{ss,max}^{* }$ for both logic gates (Fig. 4b):

$$\frac{m{G}_{ss}^{* }}{m{G}_{ss,max}^{* }}=\left\{\begin{array}{ll}\frac{{R}_{ss}^{* }}{{R}_{ss,max}^{* }}\frac{{R}_{ss,max}^{* }+c+M}{{R}_{ss}^{* }+c+{d}_{0}{k}_{feedback}^{AND}{h}_{1}(m{G}_{ss}^{* }){h}_{2}(m{G}_{ss}^{* })},\quad &\,{{\mbox{for the AND logic gate}}}\,\\ \frac{{R}_{ss}^{* }}{{R}_{ss,max}^{* }}\frac{{R}_{ss,max}^{* }+c+M}{{R}_{ss}^{* }+c+{d}_{0}{k}_{feedback}^{OR}[{h}_{1}(m{G}_{ss}^{* })+{h}_{2}(m{G}_{ss}^{* })]},\quad &\,{{\mbox{for the OR logic gate}}}\,\end{array}\right.$$

where *d*_0_ and *c* are constants. The ${k}_{feedback}^{AND}$ and ${k}_{feedback}^{OR}$ represent the negative feedback strength for the system with the AND logic gate and the system with the OR logic gate, respectively. The ${h}_{1}(m{G}_{ss}^{* })$ and ${h}_{2}(m{G}_{ss}^{* })$ describe how *mG*_*ss*_ determines *tGEF*_*ss*_ and $t{G}_{ss}^{* }$, respectively. $M={d}_{0}{k}_{feedback}^{AND}{h}_{1}(m{G}_{ss,max}^{* }){h}_{2}(m{G}_{ss,max}^{* })={d}_{0}{k}_{feedback}^{OR}[{h}_{1}(m{G}_{ss,max}^{* })+{h}_{2}(m{G}_{ss,max}^{* })]$, where the last equality originates from the constraint of the same mGTPase activation level. The only difference in the last term in the denominator cannot have a huge impact on the $m{G}_{ss}^{* }/m{G}_{ss,max}^{* }$ value.

As for the Hill-function model, the DoRA behavior for the OR logic gate is still almost the same as that for the AND logic gate (Fig. 4c). The fact that the $m{G}_{ss}^{* }/m{G}_{ss,max}^{* }$ curve for the AND logic gate is higher than that for the OR logic gate still holds (the left column in Fig. 4d), but the $m{G}_{ss}^{* }/m{G}_{ss,max}^{* }$ curve moves from the left to the right of the receptor curve with increased feedback strength in the Hill-function model (the right column in Fig. 4d). Therefore, the high mGTPase activation level (i.e., low feedback strength) results in better DoRA for the OR logic gate than the AND logic gate (dots in the red box in Fig. 4c); the low mGTPase activation level may lead to the better DoRA for the AND logic gate (dots in the pink box in Fig. 4c). But one important feature of these dots is that they are more close to the diagonal line, compared with those in the mass action model. This is because of the saturation effect of Hill function: though the mGAP levels in the model using AND or OR logic gate can differ a lot, its effect on the mGTPase, *f*_*act*_(*mGAP*), might be the same if *f*_*act*_ reaches the saturation level 1.

### DoRA in a longer cascade can be obtained using Hill-function kinetics under certain conditions

From above analyses, the DoRA behaviors for m- and tGTPases always show the same tendency, for example, when increasing mGAP level, adding negative feedback, or modeling feedback with distinct logic gates. But which GTPases have a better DoRA performance under the same circumstance? To answer this question, we compared the distance metrics between m- and tGTPases in the mass action or Hill-function model.

**Fig. 5:**
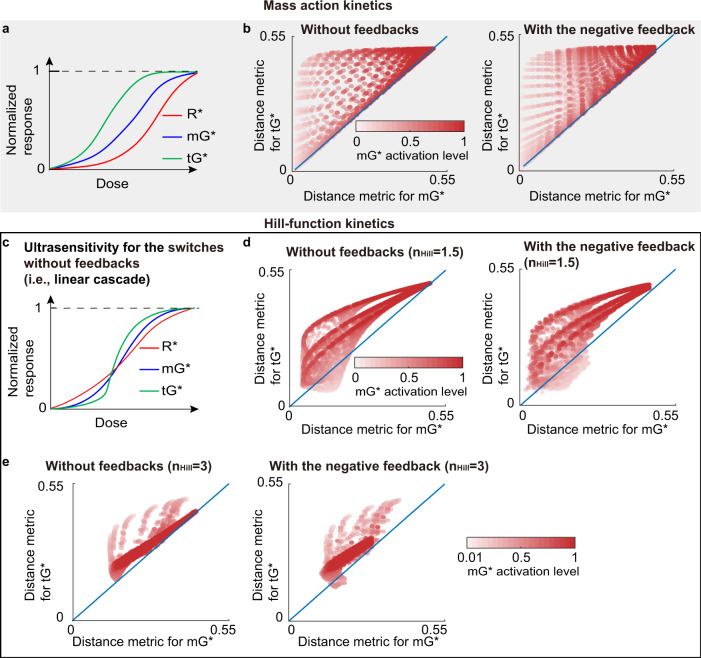
DoRA in a longer cascade can be obtained using Hill-function kinetics and low mGTPase activation level. **a** The DoRA of mGTPase is better than that of tGTPase in the mass action model. This is because $\frac{t{G}_{ss}^{* }}{t{G}_{ss,max}^{* }}\ge \frac{m{G}_{ss}^{* }}{m{G}_{ss,max}^{* }}\ge \frac{{R}_{ss}^{* }}{{R}_{ss,max}^{* }}$ holds all the time. **b** The scatter plots of distance metric for mGTPase vs tGTPase in the mass action model. Each dot corresponds to a set of kinetic parameters. The blue line is the diagonal line. The left and right panels correspond to the case without and without feedback, respectively. The number of kinetic parameter sets is one-fourth of that in Fig. 2b(3). **c** The ultrasensitivity for the circuit without feedback where the Hill-function kinetics is adopted. **d–e** Same plots as in (**b**) except that the model is Hill-function based with the Hill coefficient 1.5 (**d**) or 3 (**e**).

For the mass action model, the DoRA behavior for tGTPase is worse than that for mGTPase, suggesting that longer cascade shows worse DoRA performance. This result is validated by the following theoretical analyses (Fig. 5a). As we have shown in the previous section and Methods section, the normalized steady-state value of mG^*^ in the circuit without feedback is given by

$$\frac{m{G}_{ss}^{* }}{m{G}_{ss,max}^{* }}=\frac{{R}_{ss}^{* }}{{R}_{ss,max}^{* }}\frac{({R}_{ss,max}^{* }+{a}_{M})}{{R}_{ss}^{* }+{a}_{M}}.$$

Thus, $\frac{m{G}_{ss}^{* }}{m{G}_{ss,max}^{* }}\ge \frac{{R}_{ss}^{* }}{{R}_{ss,max}^{* }}$ all the time due to ${R}_{ss,max}^{* }\ge {R}_{ss}^{* }$. For the circuit with negative feedback loops, *a*_*M*_ becomes an increasing function of $m{G}_{ss}^{* }$, and thus this relation also holds. Using a similar way, we can prove that the $\frac{t{G}_{ss}^{* }}{t{G}_{ss,max}^{* }}\ge \frac{m{G}_{ss}^{* }}{m{G}_{ss,max}^{* }}$ all the time. Therefore, we got $\frac{t{G}_{ss}^{* }}{t{G}_{ss,max}^{* }}\ge \frac{m{G}_{ss}^{* }}{m{G}_{ss,max}^{* }}\ge \frac{{R}_{ss}^{* }}{{R}_{ss,max}^{* }}$, that is, the normalized dose-response curve of tGTPase is higher than that of mGTPase, and the latter is higher than that of the receptor. According to the locations of different curves, the tGTPase curve is farther from the receptor curve, leading to the worse DoRA. This is also supported by the scatter plots of the distance metric between mGTPase and tGTPase (Fig. 5b).

As for the Hill-function model, the better DoRA performance of tGTPase than mGTPase occurs when the mGTPase activation level is low. A widely observed property in the linear cascade is the ultrasensitivity, where the long cascade results in a steep response curve (Fig. 5c). It means that the mGTPase curve is closer to the receptor curve than the tGTPase curve, especially when the dose is very low or high. Thus, the mGTPase exhibits better DoRA behavior than the tGTPase in most cases (dots above the diagonal line in Fig. 5d and Supplementary Fig. 8f). Other cases where the tGTPase performs better may result from the complicated behavior when the dose is in the intermediate level; numerical simulations suggest that these cases may occur when the mGTPase activation level is low (dots under the diagonal line in Fig. 5d and Supplementary Fig. 8f). The underlying mechanism for why tGTPase can display a better DoRA behavior with a low mGTPase activation level remains unknown, and increasing the Hill coefficient to 3 does not seem to expand this advantage. Moreover, increasing the Hill coefficient makes the lowest value of distance metrics increase (Fig. 5d–e and Supplementary Fig. 9), indicating that a high Hill coefficient may impair DoRA.

## Discussion

**Fig. 6:**
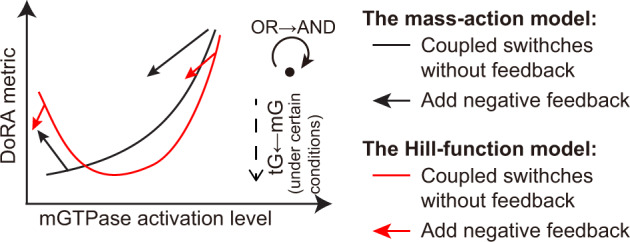
Summary of the effects of kinetic details on the DoRA. DoRA metric is closely related to mGTPase activation level, and this relation depends on reaction kinetics, the existence of negative feedbacks (straight solid arrow), cascade length (straight dashed arrow), and logics for negative feedback (curved solid arrow).

A longstanding question in biology is how cells accurately transmit information from the extracellular space and thus make right decisions. The DoRA is one of the mechanisms to ensure cells respond proportionally to the external stimulus. Though the “push-pull” topology can produce perfect DoRA^7,9^, the prevalence of the negative within DoRA motifs may indicate the possibility of the negative feedback to achieve the near-perfect DoRA under certain circumstances. We explored the design principles of DoRA in the circuit where the negative feedback loops couple two types of GTPase switches. In the absence of feedback, kinetic parameters that ensure low mGTPase activation produce good DoRA performance in the mass action model (black solid line in Fig. 6); however, an intermediate mGTPase activation level is preferred in the Hill-function model (red solid line in Fig. 6). Adding negative feedback loops cannot always enhance the DoRA performance; the enhancement role of negative feedback loops occurs for the mass action model with high mGTPase activation (the right black arrow in Fig. 6) or for the Hill-function model with high or extremely low mGTPase activation (red arrows in Fig. 6). Besides, adding negative feedback loops causes a larger change in DoRA performance in the mass action model compared with the Hill-function model, but modeling negative feedback loops *tGEF* → *mGAP* and tG^*^ → *mGAP* with the AND or OR logic gate seems to have little difference (the curved arrow in Fig. 6). Furthermore, DoRA performance in a longer cascade can be obtained using Hill-function kinetics and with a low mGTPase activation level (dashed arrow in Fig. 6).

These results suggest the emergence of different DoRA behavior when comparing the simpler mass action kinetics to the more complex (i.e., nonlinear activation rates) Hill-function kinetics. This is mainly because the Hill-function model allows more relative positions of normalized dose-response curves for GTPase and the receptor (Fig. 2c and Fig. 3c), while the mass action model only exhibits one case–the normalized dose-response curve for GTPase is higher than that for the receptor (Figs. 2a and 3a; Fig. 2a in^7^ if scaling the curve to ensure the maximal value 1). It appears that the Hill-function kinetics reveal more features than the mass action kinetics. First, the Hill-function model seems to attain DoRA more easily than the mass action model, because the biologically plausible level of the mGTPase activation cannot be extremely low, deviating the condition for good DoRA in the mass action model. Second, the Hill-function model shows a loosened requirement on mGTPase activation level to achieve the negative feedback’s role in improving DoRA than the mass action model; the experiment also observed the negative feedback’s role in DoRA^4^.

Through the analysis of three coupled molecular switches (i.e., a longer cascade), we found that the reactant in the distal switch exhibits better DoRA than that in the proximal switch only when Hill-function kinetics is adopted and the mGTPase activation level is low. This suggests another advantage of the Hill function kinetics—making the downstream reactant achieve better DoRA than the upstream reactant. This advantage ensures the information transfer in long cellular signaling pathways. Although the negative feedback loops are widely regarded to linearize the downstream reactant^4,51^, that we observed similar results for the circuit without or with feedback (the bottom panel in Fig. 3d) implies that the key factor to maintain a better DoRA for tGTPase might be the level of mGTPase activation instead of the negative feedback, providing new insight about the mechanism for good DoRA.

The highly context-dependent role of negative feedback in enhancing DoRA suggested by this work improves our understanding of negative feedback, where previous computational studies only mentioned that negative feedback loops cannot achieve perfect DoRA^9^. Interestingly, experiments in many signaling pathways^4,26^ showed that negative feedback always improves DoRA performance, which is not consistent with the versatile role of negative feedback found in this work. This inconsistency may come from the complexity of realistic signaling pathways, and we anticipate that there might exist complex kinetics where the negative feedback improves DoRA performance under any circumstance, for instance, the higher order mass action kinetics *x*^4^*y* where *x* and y are both reactants, or Hill-function kinetics with different Michaelis constants for distinct reactions. Furthermore, our analyses only considered the DoRA performance, while there are many other metrics for the efficiency of the dose information transmission. If using other metrics, the role of negative feedback may change: the negative feedback can increase or decrease the channel capacity under different conditions, thus causing the increase or decrease of information transmission^18,52^; the negative feedback decreases the variance in gene expression, leading to accurate information transmission^53,54^.

This work provides a way to tune the mGTPase activation level in order to obtain good DoRA performance. For example, reducing the mGTPase activation level in the mass action model improves DoRA, and tuning the mGTPase activation level to an intermediate range in the Hill-function model leads to good DoRA. However, these changes in the mGTPase activation level may destroy other biological functions. Specifically, affected biological functions may include secretion and cell survival, because GIV-depleted cells (GIV is the tGEF in Fig. 1c) show not only the high activity of Arf1 (the mGTPase in 1c) but also the decreased secretion and cell proliferation^34^. Therefore, the biological system might be a multi-objective optimization problem, that is, achieving good DoRA performance while maintaining other biological functions simultaneously.

Finally, this work analyzes DoRA behavior in coupled molecular switches (i.e., GTPases), in which each species is limited to just 2-states (GDP or GTP-bound). One notable distinction between DoRA in coupled switches versus other instances where phosphorylation or other such cascades are in play, is that the 2-states are directly dependent on the local concentrations of GEFs (rate limiting) and GAPs and hence, they are unlikely to be saturated in certain circumstances (e.g. saturation of enzymes in a covalent modification cycle tends to reduce information transmission). By including the GAPs and GEFs in the circuit^55^, this work ensures that the emergent features of the motif are explored while attempting to preserve the biological context.

One limitation of our studies is that we neglected the effect of basal constant production rates. The non-zero basal production rates will cause the non-zero response even under the extremely low stimulus, leading to a change of relative positions of dose-response curves. While the basal constant production rates are usually small, we anticipate that our main results will not change dramatically. Another future direction is to study the effect of the receptor number on the DoRA in the circuits we studied. In many signaling pathways^28,56–60^, different receptor abundance cannot affect the downstream response, i.e., the system is robust to the change of the receptor abundance. Therefore, analyzing the mechanism of DoRA achievement under distinct receptor numbers for the circuit with the negative feedback will be significant.

## Methods

### Numerical simulations

Numerical simulations were implemented in MATLAB. To obtain the dose-response curve for a given parameter set, we use the solver ode15s to simulate the dynamics of Eq. (1)-Eq. (6) on the time interval [0,10^8^] for the stimulus varied from 10^−5^ to 10^3^. The large time interval ensures that the steady state is reached. Matlab codes can be accessed at https://github.com/RangamaniLabUCSD/Qiao_et_al_Dose-response-alignment.

### Analysis for the relation between the mGTPase activation level and the DoRA in coupled switches without feedback

#### The mass action model

We first focused on the coupled switches without feedback that is modeled by the mass action kinetics. In this case, *F*(*tGEF*, tG^*^) = 0 and *f*(*x*) = *x*. Based on these two equations, we set the right terms of Eq. (1)-Eq. (6) equal to zeros and got the following equations:

$$\begin{array}{l}{R}_{ss}^{* }=\frac{{k}_{on}^{R}S}{{k}_{on}^{R}S+{k}_{off}},\quad mGE{F}_{ss}=\frac{{k}_{on}^{mGEF}}{{k}_{off}^{mGEF}}{R}_{ss}^{* },\quad m{G}_{ss}^{* }=\frac{{k}_{on}^{mG}mGE{F}_{ss}}{{k}_{on}^{mG}mGE{F}_{ss}+{k}_{off}^{mG}mGA{P}_{ss}},\\ tGE{F}_{ss}=\frac{{k}_{on}^{tGEF}}{{k}_{off}^{tGEF}}m{G}_{ss}^{* },\quad t{G}_{ss}^{* }=\frac{{k}_{on}^{tG}tGE{F}_{ss}}{{k}_{on}^{tG}tGE{F}_{ss}+{k}_{off}^{tG}tGAP},\quad mGA{P}_{ss}=\frac{{k}_{on}^{mGAP}}{{k}_{off}^{mGAP}},\end{array}$$

where ${R}_{ss}^{* },mGE{F}_{ss},mGA{P}_{ss},m{G}_{ss}^{* },tGE{F}_{ss},t{G}_{ss}^{* }$, denote the steady-state values of R^*^, *mGEF*, *mGAP*, mG^*^, *tGEF*, tG^*^, respectively, From these equations, we can easily get the following expressions for $m{G}_{ss}^{* }$ and $t{G}_{ss}^{* }$

9 $$m{G}_{ss}^{* }=\frac{{R}_{ss}^{* }}{{R}_{ss}^{* }+{a}_{M}},\quad t{G}_{ss}^{* }=\frac{m{G}_{ss}^{* }}{m{G}_{ss}^{* }+{b}_{M}}=\frac{1}{1+{b}_{M}}\frac{{R}_{ss}^{* }}{{R}_{ss}^{* }+\frac{{a}_{M}{b}_{M}}{1+{b}_{M}}}$$

where ${a}_{M}=mGA{P}_{ss}\frac{{k}_{off}^{mGEF}{k}_{off}^{mG}}{{k}_{on}^{mG}{k}_{on}^{mGEF}}$ and ${b}_{M}=tGAP\frac{{k}_{off}^{tGEF}{k}_{off}^{tG}}{{k}_{on}^{tG}{k}_{on}^{tGEF}}$. The maximal value of ${R}_{ss}^{* }$ is 1, which is obtained when the stimulus *S* goes to infinity, so the maximal values of $m{G}_{ss}^{* }$ and $t{G}_{ss}^{* }$ are given by:

10 $$m{G}_{ss,max}^{* }=\frac{1}{1+{a}_{M}},$$

11 $$t{G}_{ss,max}^{* }=\frac{1}{1+{b}_{M}}\frac{1}{1+\frac{{a}_{M}{b}_{M}}{1+{b}_{M}}}.$$

Therefore, the normalized $m{G}_{ss}^{* }$ and $t{G}_{ss}^{* }$ are shown as follows:

12 $$\frac{m{G}_{ss}^{* }}{m{G}_{ss,max}^{* }}=\frac{(1+{a}_{M}){R}_{ss}^{* }}{{R}_{ss}^{* }+{a}_{M}},\quad \frac{t{G}_{ss}^{* }}{t{G}_{ss,max}^{* }}={R}_{ss}^{* }\frac{1+\frac{{a}_{M}{b}_{M}}{1+{b}_{M}}}{{R}_{ss}^{* }+\frac{{a}_{M}{b}_{M}}{1+{b}_{M}}}.$$

Then, if we rewrite *a*_*M*_ as a function of $m{G}_{ss,max}^{* }$ using Eq. (10) and substitute the *a*_*M*_ in Eq. (12), we get the following relation between normalized GTPases and $m{G}_{ss,max}^{* }$:

13 $$\frac{m{G}_{ss}^{* }}{m{G}_{ss,max}^{* }}=\frac{{R}_{ss}^{* }}{1-m{G}_{ss,max}^{* }(1-{R}_{ss}^{* })},\quad \frac{t{G}_{ss}^{* }}{t{G}_{ss,max}^{* }}={R}_{ss}^{* }\frac{1+\left(\frac{1}{m{G}_{ss,max}^{* }}-1\right)\frac{{b}_{M}}{1+{b}_{M}}}{{R}_{ss}^{* }+\left(\frac{1}{m{G}_{ss,max}^{* }}-1\right)\frac{{b}_{M}}{1+{b}_{M}}}.$$

From Eq. (12) and Eq. (13), we can obtain following properties for m- and tGTPase:

- Normalized GTPase curves are always higher than the receptor curve, because Eq. (12) leads to $\frac{m{G}_{ss}^{* }}{m{G}_{ss,max}^{* }}\ge {R}_{ss}^{* }$ and $\frac{t{G}_{ss}^{* }}{t{G}_{ss,max}^{* }}\ge {R}_{ss}^{* }$;
- Decreasing the mGTPase activation level lowers the normalized GTPase curves, because Eq. (13) indicates that $\frac{m{G}_{ss}^{* }}{m{G}_{ss,max}^{* }}$ and $\frac{t{G}_{ss}^{* }}{t{G}_{ss,max}^{* }}$ both decrease with decreased $m{G}_{ss,max}^{* }$.

Taking these two properties together, we conclude that the low mGTPase activation level makes the normalized GTPase curves close to the receptor curve, i.e., good DoRA.

#### The Hill-function model

Next, for the same circuit, we turned to the Hill-function model to explore the relation between the mGTPase activation level and the DoRA behavior. Here, *f*(*x*) is Hill function, and *F*(*tGEF*, tG^*^) = 0. Let the right-hand terms of Eq. (1)-Eq. (6) equal to zeros and substitute *f* with the Hill function *f*_*act*_. Then relations between steady states are as follows:

$$\begin{array}{l}{R}_{ss}^{* }=\frac{{k}_{on}^{R}{f}_{act}(S)}{{k}_{on}^{R}{f}_{act}(S)+{k}_{off}},\quad mGE{F}_{ss}=\frac{{k}_{on}^{mGEF}}{{k}_{off}^{mGEF}}{f}_{act}({R}_{ss}^{* }),\\ m{G}_{ss}^{* }=\frac{{k}_{on}^{mG}{f}_{act}(mGE{F}_{ss})}{{k}_{on}^{mG}{f}_{act}(mGE{F}_{ss})+{k}_{off}^{mG}{f}_{act}(mGA{P}_{ss})},tGE{F}_{ss}=\frac{{k}_{on}^{tGEF}}{{k}_{off}^{tGEF}}{f}_{act}(m{G}_{ss}^{* }),\\ t{G}_{ss}^{* }=\frac{{k}_{on}^{tG}{f}_{act}(tGE{F}_{ss})}{{k}_{on}^{tG}{f}_{act}(tGE{F}_{ss})+{k}_{off}^{tG}{f}_{act}(tGAP)},\quad mGA{P}_{ss}=\frac{{k}_{on}^{mGAP}}{{k}_{off}^{mGAP}}.\end{array}$$

Based on these equations, we got the following equations for steady states of mG* and tG*:

14 $$m{G}_{ss}^{* }=\frac{{F}_{1}({R}_{ss}^{* })}{{F}_{1}({R}_{ss}^{* })+{a}_{H}},$$

15 $$t{G}_{ss}^{* }=\frac{{F}_{2}({R}_{ss}^{* })}{{F}_{2}({R}_{ss}^{* })+{b}_{H}},$$

where ${F}_{1}({R}_{ss}^{* })={f}_{act}\left(\frac{{k}_{on}^{mGEF}}{{k}_{off}^{mGEF}}{f}_{act}({R}_{ss}^{* })\right)$, ${F}_{2}({R}_{ss}^{* })={f}_{act}\left(\frac{{k}_{on}^{tGEF}}{{k}_{off}^{tGEF}}{f}_{act}\left(\frac{{F}_{1}({R}_{ss}^{* })}{{F}_{1}({R}_{ss}^{* })+{a}_{H}}\right)\right)$, ${a}_{H}=\frac{{k}_{on}^{mG}}{{k}_{off}^{mG}}{f}_{act}(mGA{P}_{ss})$, and ${b}_{H}=\frac{{k}_{on}^{tG}}{{k}_{off}^{tG}}{f}_{act}(tGAP)$. We used ${R}_{ss,max}^{* }$ to denote the value of ${R}_{ss}^{* }$ when the stimulus *S* goes to infinity. It should be noted that ${R}_{ss,max}^{* }$ is no longer 1 because the *f*_*act*_(*S*) has the maximal value 1 and thus cannot become infinity even when *S* goes to infinity. So, the maximal values of $m{G}_{ss}^{* }$ and $t{G}_{ss}^{* }$, denoted by $m{G}_{ss,max}^{* }$ and $t{G}_{ss,max}^{* }$, are given by:

16 $$m{G}_{ss,max}^{* }=\frac{{F}_{1}({R}_{s,max}^{* })}{{F}_{1}({R}_{ss,max}^{* })+{a}_{H}},$$

17 $$t{G}_{ss,max}^{* }=\frac{{F}_{2}({R}_{ss,max}^{* })}{{F}_{2}({R}_{ss,max}^{* })+{b}_{H}}.$$

Dividing the $m{G}_{ss}^{* }$ and $t{G}_{ss}^{* }$ by these two maximal values, we got the following equations:

18 $$\frac{m{G}_{ss}^{* }}{m{G}_{ss,max}^{* }}=\frac{{F}_{1}({R}_{ss}^{* })}{{F}_{1}({R}_{ss,max}^{* })}\frac{{F}_{1}({R}_{ss,max}^{* })+{a}_{H}}{{F}_{1}({R}_{ss}^{* })+{a}_{H}},$$

19 $$\frac{t{G}_{ss}^{* }}{t{G}_{ss,max}^{* }}=\frac{{F}_{2}({R}_{ss}^{* })}{{F}_{2}({R}_{ss,max}^{* })}\frac{{F}_{2}({R}_{ss,max}^{* })+{b}_{H}}{{F}_{2}({R}_{ss}^{* })+{b}_{H}}.$$

For the DoRA of the mGTPase, tuning one kinetic parameter while fixing other kinetic parameters to reduce the mGTPase level usually leads to the decrease of $\frac{m{G}_{ss}^{* }}{m{G}_{ss,max}^{* }}$. The proof for this conclusion depends on which kinetic parameter is chosen to tune. If ${k}_{on}^{mG}$, ${k}_{off}^{mG}$, or *mGAP*_*ss*_ is chosen, every term in Eq. (14) and Eq. (18) is unchanged except *a*_*H*_; therefore, increasing *a*_*H*_, i.e., decreasing mGTPase activation level $m{G}_{ss,max}^{* }$, causes the decrease of $\frac{m{G}_{ss}^{* }}{m{G}_{ss,max}^{* }}$. On the other hand, if ${k}_{on}^{mGEF}$, or ${k}_{off}^{mGEF}$ is chosen to tune, the function *F*_1_ in Eq. (14) and Eq. (18) changes. The way to tune ${k}_{on}^{mGEF}$ (or ${k}_{off}^{mGEF}$) in order to lower the mGTPase activation level is decreasing ${k}_{on}^{mGEF}$ (or increasing ${k}_{off}^{mGEF}$), leading to the decrease of $\frac{{k}_{on}^{mGEF}}{{k}_{off}^{mGEF}}$. For simplicity, we used *k* to denote $\frac{{k}_{on}^{mGEF}}{{k}_{off}^{mGEF}}$. Then we proved that the decrease of *k* results in the decrease of the $\frac{m{G}_{ss}^{* }}{m{G}_{ss,max}^{* }}$ as follows. First, from Eq. (18) the derivative of $\frac{m{G}_{ss}^{* }}{m{G}_{ss,max}^{* }}$ with respect to the *k* (i.e., $\frac{{k}_{on}^{mGEF}}{{k}_{off}^{mGEF}}$) is given by:

20 $$\begin{array}{rcl}\frac{\partial }{\partial k}\left(\frac{m{G}_{ss}^{* }}{m{G}_{ss,max}^{* }}\right)&=&\frac{\partial }{\partial k}\left(\frac{1+\frac{{a}_{H}}{{F}_{1}({R}_{ss,max}^{* })}}{1+\frac{{a}_{H}}{{F}_{1}({R}_{ss}^{* })}}\right)\\ &\propto &-\frac{1}{{F}_{1}^{2}({R}_{ss,max}^{* })}\frac{\partial {F}_{1}({R}_{ss,max}^{* })}{\partial k}\left(1+\frac{{a}_{H}}{{F}_{1}({R}_{ss}^{* })}\right)+\frac{1}{{F}_{1}^{2}({R}_{ss}^{* })}\frac{\partial {F}_{1}({R}_{ss}^{* })}{\partial k}\left(1+\frac{{a}_{H}}{{F}_{1}({R}_{ss,max}^{* })}\right)\\ &=&\underbrace{\frac{1}{{F}_{1}^{2}({R}_{ss}^{* })}\frac{\partial {F}_{1}({R}_{ss}^{* })}{\partial k}-\frac{1}{{F}_{1}^{2}({R}_{ss,max}^{* })}\frac{\partial {F}_{1}({R}_{ss,max}^{* })}{\partial k}}_{\begin{array}{c}{Q}_{1}\end{array}}\\ &&+\frac{{a}_{H}}{{F}_{1}({R}_{ss,max}^{* }){F}_{1}({R}_{ss}^{* })}\underbrace{\left(\frac{1}{{F}_{1}({R}_{ss}^{* })}\frac{\partial {F}_{1}({R}_{ss}^{* })}{\partial k}-\frac{1}{{F}_{1}({R}_{ss,max}^{* })}\frac{\partial {F}_{1}({R}_{ss,max}^{* })}{\partial k}\right)}_{\begin{array}{c}{Q}_{2}\end{array}}\end{array}$$

Then, according to the definition of *f*_*act*_, the $\frac{1}{{F}_{1}^{2}({R}_{ss}^{* })}\frac{\partial {F}_{1}({R}_{ss}^{* })}{\partial k}$ and $\frac{1}{{F}_{1}({R}_{ss}^{* })}\frac{\partial {F}_{1}({R}_{ss}^{* })}{\partial k}$ can be rewritten as follows:

$$\frac{1}{{F}_{1}^{2}({R}_{ss}^{* })}\frac{\partial {F}_{1}({R}_{ss}^{* })}{\partial k}=\frac{{[{(\frac{K}{k})}^{n}+{f}_{act}^{n}({R}_{ss}^{* })]}^{2}}{{({f}_{act}^{n}({R}_{ss}^{* }))}^{2}}\frac{{f}_{act}^{\,n}({R}_{ss}^{* })}{{[{(\frac{K}{k})}^{n}+{f}_{act}^{n}({R}_{ss}^{* })]}^{2}}\frac{n}{{k}^{2}}{\left(\frac{K}{k}\right)}^{n-1}=\frac{1}{{f}_{act}^{\,n}({R}_{ss}^{* })}\frac{n}{{k}^{2}}{\left(\frac{K}{k}\right)}^{n-1}$$

$$\frac{1}{{F}_{1}({R}_{ss}^{* })}\frac{\partial {F}_{1}({R}_{ss}^{* })}{\partial k}=\frac{{(\frac{K}{k})}^{n}+{f}_{act}^{\,n}({R}_{ss}^{* })}{{f}_{act}^{\,n}({R}_{ss}^{* })}\frac{{f}_{act}^{\,n}({R}_{ss}^{* })}{{[{(\frac{K}{k})}^{n}+{f}_{act}^{n}({R}_{ss}^{* })]}^{2}}\frac{n}{{k}^{2}}{\left(\frac{K}{k}\right)}^{n-1}=\frac{1}{{(\frac{K}{k})}^{n}+{f}_{act}^{\,n}({R}_{ss}^{* })}\frac{n}{{k}^{2}}{\left(\frac{K}{k}\right)}^{n-1}$$

Thus, $\frac{1}{{F}_{1}^{2}({R}_{ss}^{* })}\frac{\partial {F}_{1}({R}_{ss}^{* })}{\partial k}$ and $\frac{1}{{F}_{1}({R}_{ss}^{* })}\frac{\partial {F}_{1}({R}_{ss}^{* })}{\partial k}$ are both decreasing function of ${R}_{ss}^{* }$, and thus *Q*_1_ > 0 and *Q*_2_ > 0. So the $\frac{\partial }{\partial k}\left(\frac{m{G}_{ss}^{* }}{m{G}_{ss,max}^{* }}\right)$ in Eq. (20) is larger than 0, finishing our proof about why decreasing *k* results in the decrease of the $\frac{m{G}_{ss}^{* }}{m{G}_{ss,max}^{* }}$.

As for the DoRA of tGTPase, the $\frac{t{G}_{ss}^{* }}{t{G}_{ss,max}^{* }}$ is also positively correlated with the mGTPase activation level if only kinetic parameter is varied. As we can see from the above paragraph, the kinetic parameters contributing to the mGTPase activation level appear in *F*_1_ and *a*_*H*_, and thus affect $t{G}_{ss}^{* }$ only though *F*_2_. First we studied how increasing *a*_*H*_, i.e., decreasing the mGTPase activation level, influences the $\frac{t{G}_{ss}^{* }}{t{G}_{ss,max}^{* }}$. Similarly to the case of mGTPase, the sign of $\frac{\partial }{\partial {a}_{H}}\left(\frac{t{G}_{ss}^{* }}{t{G}_{ss,max}^{* }}\right)$ is determined by $\frac{1}{{F}_{2}^{2}({R}_{ss}^{* })}\frac{\partial {F}_{2}({R}_{ss}^{* })}{\partial {a}_{H}}-\frac{1}{{F}_{2}^{2}({R}_{ss,max}^{* })}\frac{\partial {F}_{2}({R}_{ss,max}^{* })}{\partial {a}_{H}}$ and $\frac{1}{{F}_{2}({R}_{ss}^{* })}\frac{\partial {F}_{2}({R}_{ss}^{* })}{\partial {a}_{H}}-\frac{1}{{F}_{2}({R}_{ss,max}^{* })}\frac{\partial {F}_{2}({R}_{ss,max}^{* })}{\partial {a}_{H}}$. Combined with the definition of ${F}_{2}({R}_{ss}^{* })$, the $\frac{1}{{F}_{2}^{2}({R}_{ss}^{* })}\frac{\partial {F}_{2}({R}_{ss}^{* })}{\partial {a}_{H}}$ and $\frac{1}{{F}_{2}({R}_{ss}^{* })}\frac{\partial {F}_{2}({R}_{ss}^{* })}{\partial {a}_{H}}$ can be rewritten as:

$$\begin{array}{ll}\frac{1}{{F}_{2}^{2}({R}_{ss}^{* })}\frac{\partial {F}_{2}({R}_{ss}^{* })}{\partial {a}_{H}}\,=\,-\,\frac{{[{(\frac{K}{{k}_{t}})}^{n}+{f}_{act}^{n}(Y)]}^{2}}{{({f}_{act}^{n}(Y))}^{2}}\frac{n{(\frac{K}{{k}_{t}})}^{n}{f}_{act}^{\,n-1}(Y)}{{[{(\frac{K}{{k}_{t}})}^{n}+{f}_{act}^{n}(Y)]}^{2}}\frac{n{K}^{n}{f}_{act}(Y)}{Y({Y}^{n}+{K}^{n})}\frac{Y}{{F}_{1}({R}_{ss}^{* })+{a}_{H}}\\ \qquad\qquad\qquad\quad\propto -\,\frac{1}{{f}_{act}^{\,n}(Y)}\frac{1}{({Y}^{n}+{K}^{n})}\frac{1}{{F}_{1}({R}_{ss}^{* })+{a}_{H}},\end{array}$$

$$\begin{array}{ll}\frac{1}{{F}_{2}({R}_{ss}^{* })}\frac{\partial {F}_{2}({R}_{ss}^{* })}{\partial {a}_{H}}\,=\,-\,\frac{{(\frac{K}{{k}_{t}})}^{n}+{f}_{act}^{\,n}(Y)}{{f}_{act}^{\,n}(Y)}\frac{n{(\frac{K}{{k}_{t}})}^{n}{f}_{act}^{\,n-1}(Y)}{{[{(\frac{K}{{k}_{t}})}^{n}+{f}_{act}^{n}(Y)]}^{2}}\frac{n{K}^{n}{f}_{act}(Y)}{Y({Y}^{n}+{K}^{n})}\frac{Y}{{F}_{1}({R}_{ss}^{* })+{a}_{H}}\\ \qquad\qquad\qquad\quad\propto -\,\frac{1}{{f}_{act}^{\,n}(Y)+{(\frac{K}{{k}_{t}})}^{n}}\frac{1}{({Y}^{n}+{K}^{n})}\frac{1}{{F}_{1}({R}_{ss}^{* })+{a}_{H}}\end{array}$$

where $Y=\frac{{F}_{1}({R}_{ss}^{* })}{{F}_{1}({R}_{ss}^{* })+{a}_{H}}$ and ${k}_{t}=\frac{{k}_{on}^{tGEF}}{{k}_{off}^{tGEF}}$. So $\frac{1}{{F}_{2}^{2}({R}_{ss}^{* })}\frac{\partial {F}_{2}({R}_{ss}^{* })}{\partial {a}_{H}}$ and $\frac{1}{{F}_{2}({R}_{ss}^{* })}\frac{\partial {F}_{2}({R}_{ss}^{* })}{\partial {a}_{H}}$ are both increasing function of ${R}_{ss}^{* }$, leading the negative sign of the derivative of $\frac{\partial }{\partial {a}_{H}}(\frac{t{G}_{ss}^{* }}{t{G}_{ss,max}^{* }})$ with respect to the *a*_*H*_. This indicates that the large *a*_*H*_ not only decreases the mGTPase activation level but also lowers the $\frac{\partial }{\partial {a}_{H}}(\frac{t{G}_{ss}^{* }}{t{G}_{ss,max}^{* }})$. For the case where only *k* is varied, decreasing *k* causes both the low mGTPase activation level and the low $\frac{t{G}_{ss}^{* }}{t{G}_{ss,max}^{* }}$, because $\frac{1}{{F}_{2}^{2}({R}_{ss}^{* })}\frac{\partial {F}_{2}({R}_{ss}^{* })}{\partial k}$ and $\frac{1}{{F}_{2}({R}_{ss}^{* })}\frac{\partial {F}_{2}({R}_{ss}^{* })}{\partial k}$ are both decreasing functions of ${R}_{ss}^{* }$ as shown below:

$$\begin{array}{l}\frac{1}{{F}_{2}^{2}({R}_{ss}^{* })}\frac{\partial {F}_{2}({R}_{ss}^{* })}{\partial k}\\ \,=\,\frac{{[{(\frac{K}{{k}_{t}})}^{n}+{f}_{act}^{n}(Y)]}^{2}}{{({f}_{act}^{n}(Y))}^{2}}\frac{n{(\frac{K}{{k}_{t}})}^{n}{f}_{act}^{\,n-1}(Y)}{{[{(\frac{K}{{k}_{t}})}^{n}+{f}_{act}^{n}(Y)]}^{2}}\frac{n{K}^{n}{f}_{act}(Y)}{Y({Y}^{n}+{K}^{n})}\frac{{a}_{H}Y}{{F}_{1}({R}_{ss}^{* })+{a}_{H}}\frac{1}{{(\frac{K}{k})}^{2}+{f}_{act}^{n}({R}_{ss}^{* })}\frac{n}{{k}^{2}}{(\frac{K}{k})}^{n-1}\\ \propto \frac{1}{{f}_{act}^{\,n}(Y)}\frac{1}{({Y}^{n}+{K}^{n})}\frac{1}{{F}_{1}({R}_{ss}^{* })+{a}_{H}}\frac{1}{{(\frac{K}{k})}^{2}+{f}_{act}^{n}({R}_{ss}^{* })},\end{array}$$

$$\begin{array}{ll}\frac{1}{{F}_{2}({R}_{ss}^{* })}\frac{\partial {F}_{2}({R}_{ss}^{* })}{\partial k}\\ \,=\,\frac{{(\frac{K}{{k}_{t}})}^{n}+{f}_{act}^{\,n}(Y)}{{f}_{act}^{\,n}(Y)}\frac{n{(\frac{K}{{k}_{t}})}^{n}{f}_{act}^{\,n-1}(Y)}{{[{(\frac{K}{{k}_{t}})}^{n}+{f}_{act}^{n}(Y)]}^{2}}\frac{n{K}^{n}{f}_{act}(Y)}{Y({Y}^{n}+{K}^{n})}\frac{{a}_{H}Y}{{F}_{1}({R}_{ss}^{* })+{a}_{H}}\frac{1}{{(\frac{K}{k})}^{2}+{f}_{act}^{n}({R}_{ss}^{* })}\frac{n}{{k}^{2}}{(\frac{K}{k})}^{n-1}\\ \propto \frac{1}{{f}_{act}^{\,n}(Y)+{(\frac{K}{{k}_{t}})}^{n}}\frac{1}{({Y}^{n}+{K}^{n})}\frac{1}{{F}_{1}({R}_{ss}^{* })+{a}_{H}}\frac{1}{{(\frac{K}{k})}^{2}+{f}_{act}^{n}({R}_{ss}^{* })}.\end{array}$$

The above analyses showed that tuning one kinetic parameter to achieve low mGTPase activation level leads to the low $\frac{m{G}_{ss}^{* }}{m{G}_{ss,max}^{* }}$ and $\frac{t{G}_{ss}^{* }}{t{G}_{ss,max}^{* }}$. So, the $\frac{m{G}_{ss}^{* }}{m{G}_{ss,max}^{* }}$ or $\frac{t{G}_{ss}^{* }}{t{G}_{ss,max}^{* }}$ crosses the receptor curve from the left to the right. This observation suggests the distance metric experiences a decreasing and increasing trend (Fig. 2c). It should be noted that we didn’t consider the effects of changing kinetic parameters *B* or *EC*_50_ in the function *f*_*act*_.

### Analysis for the feedback’s effect on the DoRA

#### The mass action model

We first analyzed how the DoRA is affected by the negative feedback in the mass action model. The steady state of the mGAP concentration is given by

$$mGA{P}_{ss}=\frac{{k}_{on}^{mGAP}}{{k}_{off}^{mGAP}}+\frac{{k}_{feedback}}{{k}_{off}^{mGAP}}tGE{F}_{ss}\cdot t{G}_{ss}^{* }$$

for the AND logic gate, and

$$mGA{P}_{ss}=\frac{{k}_{on}^{mGAP}}{{k}_{off}^{mGAP}}+\frac{{k}_{feedback}}{{k}_{off}^{mGAP}}\left(tGE{F}_{ss}+t{G}_{ss}^{* }\right)$$

for the OR logic gate. Therefore, the steady state of the mG^*^ can be written as:

$$m{G}_{ss}^{* }=\frac{{R}_{ss}^{* }}{{R}_{ss}^{* }+c+h(m{G}_{ss}^{* },{k}_{feedback})},$$

where

$$h(m{G}_{ss}^{* },{k}_{feedback})=\left\{\begin{array}{ll}{k}_{feedback}\cdot d\frac{{m{G}_{ss}^{* }}^{2}}{m{G}_{ss}^{* }+{b}_{M}}\quad &\,{{\mbox{for the AND logic gate}}}\,\\ {k}_{feedback}\left(d\cdot m{G}_{ss}^{* }+e\frac{m{G}_{ss}^{* }}{m{G}_{ss}^{* }+{b}_{M}}\right)\quad &\,{{\mbox{for the OR logic gate}}}\,\end{array}\right.$$

Here, *c*, *d*, *e*, *b*_*M*_ are constants and defined as follows: $c=\frac{{k}_{on}^{mGAP}{k}_{off}^{mGEF}{k}_{off}^{mG}}{{k}_{off}^{mGAP}{k}_{on}^{mG}{k}_{on}^{mGEF}}$, $d=\frac{{k}_{on}^{tGEF}{k}_{off}^{mGEF}{k}_{off}^{mG}}{{k}_{off}^{mGAP}{k}_{off}^{tGEF}{k}_{on}^{mG}{k}_{on}^{mGEF}}$, $e=\frac{{k}_{off}^{mGEF}{k}_{off}^{mG}}{{k}_{off}^{mGAP}{k}_{on}^{mG}{k}_{on}^{mGEF}}$, and ${b}_{M}=tGAP\frac{{k}_{off}^{tG}{k}_{off}^{tGEF}}{{k}_{on}^{tG}{k}_{on}^{tGEF}}$. Note that the $\frac{\partial h}{\partial {k}_{feedback}}=\frac{h}{{k}_{feedback}}$ holds for any logic gate. For simplicity, we used *x* and *y* to denote the ${R}_{ss}^{* }$ and $m{G}_{ss}^{* }$, respectively. Then relation between ${R}_{ss}^{* }$ and $m{G}_{ss}^{* }$ can be rewritten as:

$$y=\frac{x}{x+c+h(y,{k}_{feedback})}.$$

In order to know the normalized fractional activation change when increasing feedback strength, we used *y*_*max*_ representing the maximal value of *y* when *x* is 1, and derived the expression of $\frac{\partial (y/{y}_{mx})}{\partial {k}_{feedback}}$ as follows:

$$\frac{\partial }{\partial {k}_{feedback}}\left(\frac{y}{{y}_{max}}\right)=\frac{\frac{1}{y}\frac{\partial y}{\partial {k}_{feedback}}-\frac{1}{{y}_{max}}\frac{\partial {y}_{max}}{\partial {k}_{feedback}}}{\frac{{y}_{max}}{y}}$$

Since the denominator is positive, the sign of the $\frac{\partial (y/{y}_{mx})}{\partial {k}_{feedback}}$ is only determined by the monotonicity of the function $\frac{1}{y}\frac{\partial y}{\partial {k}_{feedback}}$. Then we rewrote this function as follows:

21 $$\begin{array}{ll}\frac{1}{y}\frac{\partial y}{\partial {k}_{feedback}}\mathop{=}\limits^{M}-\,\frac{1}{y}\frac{x\frac{\partial h}{\partial {k}_{feedback}}}{x\frac{\partial h}{\partial y}+{[x+c+h(y,{k}_{feedback})]}^{2}}\\ \qquad\qquad\,=\,-\,\frac{1}{{k}_{feedback}}\frac{1}{y}\frac{xh(y,{k}_{feedback})}{x\frac{\partial h}{\partial y}+{[x+c+h(y,{k}_{feedback})]}^{2}}\\\qquad\qquad \mathop{=}\limits^{N}-\frac{1}{{k}_{feedback}}\frac{1}{y}\frac{1}{\frac{1}{h(y,{k}_{feedback})}\frac{\partial h}{\partial y}+\frac{1}{xh(y,{k}_{feedback})}{\left(\frac{x}{y}\right)}^{2}}\\ \qquad\qquad\,=\,-\,\frac{1}{{k}_{feedback}}\frac{1}{\frac{y}{h(y,{k}_{feedback})}\frac{\partial h}{\partial y}+\frac{x}{yh(y,{k}_{feedback})}}\\ \qquad\qquad\mathop{=}\limits^{P}-\,\frac{1}{{k}_{feedback}}\frac{1}{\frac{y}{h(y,{k}_{feedback})}\frac{\partial h}{\partial y}+\frac{[c+h(y,{k}_{feedback})]y}{1-y}\frac{1}{yh(y,{k}_{feedback})}}\\ \qquad\qquad\,=\,-\frac{1}{{k}_{feedback}}\frac{1}{\frac{y}{h(y,{k}_{feedback})}\frac{\partial h}{\partial y}+\frac{c+h(y,{k}_{feedback})}{h(y,{k}_{feedback})}\frac{1}{(1-y)}}.\end{array}$$

Here, the equation *M* is obtained because $\frac{\partial y}{\partial {k}_{feedback}}=-\frac{\frac{\partial H}{\partial {k}_{feedback}}}{\frac{\partial H}{\partial y}}$ where $H=\frac{x}{x+c+h(y,{k}_{feedback})}-y$. The equation *N* is based on the relation between *x* and *y*, i.e., $y=\frac{x}{x+c+h(y,{k}_{feedback})}$. This relation can also be rewritten as $x=\frac{[c+h(y,{k}_{feedback})]y}{1-y}$, which leads to the equation *P*.

From the above analysis, we showed that the sign of the $\frac{\partial (m{G}_{ss}^{* }/m{G}_{ss,max}^{* })}{\partial {k}_{feedback}}$ is determined by the difference of values of the function $\frac{y}{h(y,{k}_{feedback})}\frac{\partial h}{\partial y}+\frac{c+h(y,{k}_{feedback})}{h(y,{k}_{feedback})}\frac{1}{(1-y)}$ at $y=m{G}_{ss}^{* }$ and $y=m{G}_{ss,max}^{* }$. If this function at $m{G}_{ss}^{* }$ is smaller than that at $m{G}_{ss,max}^{* }$, the sign of the $\frac{\partial (m{G}_{ss}^{* }/m{G}_{ss,max}^{* })}{\partial {k}_{feedback}}$ is negative; however, the larger value at $m{G}_{ss}^{* }$ than that at $m{G}_{ss,max}^{* }$ indicates the positive sign. Then we focused on the monotonicity of this function with respect to *y*. For simplicity, we used *h*(*y*) to represent *h*(*y*, *k*_*feedback*_). The first term $\frac{y}{h(y)}\frac{\partial h(y)}{\partial y}$, i.e., $\frac{\partial \,{{\mbox{ln}}}h(y)}{\partial {{\mbox{ln}}}\,y}$, reflects the kinetic order: if *h*(*y*) is of second kinetic order in *y*, i.e., *h*(*y*) = *y*^2^, then $\frac{\partial \,{{\mbox{ln}}}h(y)}{\partial {{\mbox{ln}}}\,y}=2$. In fact, in our model, $\frac{y}{h(y)}\frac{\partial h(y)}{\partial y}=1+\frac{{b}_{M}}{y+{b}_{M}}$ for the AND logic gate, so the first term is an increasing function all the time; but for the OR logic gate, $\frac{\partial }{\partial y}\left(\frac{y}{h(y)}\frac{\partial h(y)}{\partial y}\right)=-e\frac{d({b}_{M}^{2}-{y}^{2})+e{b}_{M}}{{(y+{b}_{M})}^{2}{(dy+d{b}_{M}+e)}^{2}}$, so the function $\frac{y}{h(y)}\frac{\partial h(y)}{\partial y}$ decreases and then increases if *y* is larger enough. But this function seems to be flat in most range of *y* (Supplementary Figs. 4–7). The second term $\frac{1}{1-y}\frac{c+h(y)}{h(y)}$ can be approximated by $\frac{1}{1-y}$ when *y* is large, because the basal production rate of mGAP ${k}_{on}^{mGAP}$ is small and thus the constant *c* can be neglected. Therefore, the second term is an increasing function when *y* is large. In fact, when *y* is small, the existence of the $\frac{c+h(y)}{h(y)}$ makes the whole function decrease with increased *y*. The rigorous derivation of the monotonicity of the second term is as follows: 1) the derivative of this function is $\frac{\partial }{\partial y}\left(\frac{1}{1-y}\frac{c+h(y)}{h(y)}\right)=\frac{1}{{(1-y)}^{2}}\left(\frac{c+h(y)}{h(y)}-c(1-y)\frac{\partial h(y)}{\partial y}/h{(y)}^{2}\right)$; 2) the derivative of the function in the bracket is $\frac{\partial }{\partial y}\left(\frac{c+h(y)}{h(y)}-c(1-y)\frac{\partial h(y)}{\partial y}/h{(y)}^{2}\right)=-c(1-y)\frac{\partial }{\partial y}\left(\frac{\frac{\partial h}{\partial y}}{{h}^{2}(y)}\right)$; 3) $\frac{\partial }{\partial y}\left(\frac{\frac{\partial h}{\partial y}}{{h}^{2}(y)}\right)=\frac{\partial (1/h(y))}{\partial y}\left(\frac{\frac{\partial h}{\partial y}}{h(y)}\right)+\frac{1}{h(y)}\frac{\partial }{\partial y}\left(\frac{\frac{\partial h}{\partial y}}{h(y)}\right)$ is smaller than 0, because $\frac{\partial (1/h(y))}{\partial y}\le 0$, $\frac{\partial }{\partial y}\left(\frac{\frac{\partial h}{\partial y}}{h(y)}\right)=-\frac{1}{{y}^{2}}-{b}_{M}\frac{2y+{b}_{M}}{{y}^{2}{(y+{b}_{M})}^{2}}\le 0$ for the AND logic gate, and $\frac{\partial }{\partial y}\left(\frac{\frac{\partial h}{\partial y}}{h(y)}\right)=\frac{1}{h{(y)}^{2}}\left(-\frac{e{b}_{M}}{{(x+{b}_{M})}^{3}}h(y)-{(\frac{\partial h}{\partial y})}^{2}\right)\le 0$ for the OR logic. 4) so the function $-c(1-y)\frac{\partial }{\partial y}\left(\frac{\frac{\partial h}{\partial y}}{{h}^{2}(y)}\right)$ is always larger than 0, and thus the function $\frac{c+h(y)}{h(y)}-c(1-y)\frac{\partial h(y)}{\partial y}/h{(y)}^{2}$ increases monotonically with respect to *y*; 5) combining facts that the function $\frac{c+h(y)}{h(y)}-c(1-y)\frac{\partial h(y)}{\partial y}/h{(y)}^{2}$ is smaller than 0 at *y* = 0 and larger than 0 when *y* = 1, this function is negative and then becomes positive with increased *y*; 6) as the sign of this function determines the trend of the function $\frac{1}{1-y}\frac{c+h(y)}{h(y)}$, it can be concluded that the function $\frac{1}{1-y}\frac{c+h(y)}{h(y)}$ will decrease and then increase when *y* increases. Recall that the first term in $\frac{y}{h(y,{k}_{feedback})}\frac{\partial h}{\partial y}+\frac{c+h(y,{k}_{feedback})}{h(y,{k}_{feedback})}\frac{1}{(1-y)}$ is almost flat, the whole function trend is mainly determined by the second term. So, if $m{G}_{ss,max}^{* }$ is large and located in the increasing branch, the function value at $m{G}_{ss}^{* }$ has a large probability to be smaller than that at $m{G}_{ss,max}^{* }$, leading to the negative sign of $\frac{\partial }{\partial {k}_{feedback}}\frac{m{G}_{ss}^{* }}{m{G}_{ss,max}^{* }}$. However, if $m{G}_{ss,max}^{* }$ is very small which is located in the decreasing branch, the value of the function at $m{G}_{ss}^{* }$ is larger than that at $m{G}_{ss,max}^{* }$, resulting the positive sign of the $\frac{\partial }{\partial {k}_{feedback}}\frac{m{G}_{ss}^{* }}{m{G}_{ss,max}^{* }}$.

In all, the high (or low) level fo mGTPase activation may lead to the descent (or ascent) of the $\frac{m{G}_{ss}^{* }}{m{G}_{ss,max}^{* }}$ when increasing feedback strength. Based on the fact that $m{G}_{ss}^{* }/m{G}_{ss,max}^{* }$ curve is always higher than the receptor curve (due to ${R}_{ss}^{* }\frac{1+c+d\frac{{m{G}_{ss,max}^{* }}^{2}}{m{G}_{ss,max}^{* }+{b}_{M}}}{{R}_{ss}^{* }+c+d\frac{{m{G}_{ss}^{* }}^{2}}{m{G}_{ss}^{* }+{b}_{M}}}\ge {R}_{ss}^{* }$), the descent and ascent of the $\frac{m{G}_{ss}^{* }}{m{G}_{ss,max}^{* }}$ correspond to the improvement and impairment of the DoRA, respectively. Therefore, when mGTPase activation level is high, the feedback enhances the DoRA of mGTPase; such DoRA is impaired by the feedback if mGTPase activation level is low. We also compared the derivatives of the distance with respect to the feedback strength from numerical simulations and analytical analysis; they have a godd fit (Supplementary Figures 4–7). Up to now, we finished the validation of the effect of feedback on the DoRA of mGTPases.

Next we explored the role of the feedback on the DoRA of tGTPase. The steady state of tG^*^ is given by:

$$t{G}_{ss}^{* }=\frac{m{G}_{ss}^{* }}{m{G}_{ss}^{* }+{b}_{M}}=\frac{y}{y+{b}_{M}}.$$

where the constant *b*_*M*_ has been defined in the previous section. The *y* is still used to denote $m{G}_{ss}^{* }$. Similarly, we still focused on the $\frac{\partial }{\partial {k}_{feedback}}\left(\frac{t{G}_{ss}^{* }}{t{G}_{ss,max}^{* }}\right)$, that is, $\frac{\partial }{\partial {k}_{feedback}}\left(\frac{y/(y+{b}_{M})}{{y}_{max}/({y}_{max}+{b}_{M})}\right)$. This function can be rewritten as:

$$\begin{array}{rcl}&&\frac{\partial }{\partial {k}_{feedback}}\left(\frac{y/(y+{b}_{M})}{{y}_{max}/({y}_{max}+{b}_{M})}\right)\\ &=&\frac{\frac{{y}_{max}}{{y}_{max}+{b}_{M}}\,\frac{\partial }{\partial {k}_{feedback}}\left(\frac{y}{y+{b}_{M}}\right)-\frac{y}{y+{b}_{M}}\,\frac{\partial }{\partial {k}_{feedback}}\left(\frac{{y}_{max}}{{y}_{max}+{b}_{M}}\right)}{{\left(\frac{{y}_{max}}{{y}_{max}+{b}_{M}}\right)}^{2}}\\ &=&\frac{\frac{{y}_{max}}{{y}_{max}+{b}_{M}}\,\frac{{b}_{M}}{{(y+{b}_{M})}^{2}}\,\frac{\partial y}{\partial {k}_{feedback}}-\frac{y}{y+{b}_{M}}\frac{{b}_{M}}{{({y}_{max}+{b}_{M})}^{2}}\,\frac{\partial {y}_{max}}{\partial {k}_{feedback}}}{{\left(\frac{{y}_{max}}{{y}_{max}+{b}_{M}}\right)}^{2}}\\ &=&\frac{\left[\frac{1}{y}\frac{{b}_{M}}{y+{b}_{M}}\,\frac{\partial y}{\partial {k}_{feedback}}-\frac{1}{{y}_{max}}\frac{{b}_{M}}{{y}_{max}+{b}_{M}}\,\frac{\partial {y}_{max}}{\partial {k}_{feedback}}\right]\frac{y}{y+{b}_{M}}}{\frac{{y}_{max}}{{y}_{max}+{b}_{M}}}\end{array}$$

So the sign of the $\frac{\partial }{\partial {k}_{feedback}}\left(\frac{t{G}_{ss}^{* }}{t{G}_{ss,max}^{* }}\right)$ is determined by the difference of the values of the function $\frac{1}{y}\frac{{b}_{M}}{y+{b}_{M}}\frac{\partial y}{\partial {k}_{feedback}}$ at $y=m{G}_{ss}^{* }$ and $y=m{G}_{ss,max}^{* }$. Based on our previous analysis about the $\frac{1}{y}\frac{\partial y}{\partial {k}_{feedback}}$, we had $\frac{1}{y}\frac{\partial y}{\partial {k}_{feedback}} < \frac{1}{{y}_{max}}\frac{\partial {y}_{max}}{\partial {k}_{feedback}} < 0$ for the high mGTPase activation level and $\frac{1}{{y}_{max}}\frac{\partial {y}_{max}}{\partial {k}_{feedback}} < \frac{1}{y}\frac{\partial y}{\partial {k}_{feedback}} < 0$ for the low mGTPase activation level. Therefore, for the high mGTPase activation level, the $\frac{1}{y+{b}_{M}}\frac{1}{y}\frac{\partial y}{\partial {k}_{feedback}}$ is smaller than $\frac{1}{{y}_{max}+{b}_{M}}\frac{1}{{y}_{max}}\frac{\partial {y}_{max}}{\partial {k}_{feedback}}$ due to $\frac{1}{y+{b}_{M}} > \frac{1}{{y}_{max}+{b}_{M}}$; for the low mGTPase activation level, $\frac{1}{y+{b}_{M}}\frac{1}{y}\frac{\partial y}{\partial {k}_{feedback}}$ might maintain a larger value than $\frac{1}{{y}_{max}+{b}_{M}}\frac{1}{{y}_{max}}\frac{\partial {y}_{max}}{\partial {k}_{feedback}}$. Besides, because $\frac{m{G}_{ss}^{* }}{m{G}_{ss,max}^{* }}\frac{m{G}_{ss,max}^{* }+{b}_{M}}{m{G}_{ss}^{* }+{b}_{M}}\ge \frac{m{G}_{ss}^{* }}{m{G}_{ss,max}^{* }}\ge {R}_{ss}^{* }$, the $t{G}_{ss}^{* }/t{G}_{ss,max}^{* }$ curve is always higher than the receptor curve. Taken together, the DoRA of tGTPase is like that of mGTPase, i.e., the high (or low) mGTPase activation level indicates the feedback’s positive (or negative) role on the DoRA.

#### The Hill-function model

In this section, we focused on the feedback’s role in the DoRA for the Hill-function model. The steady state of the mGAP concentration is given by

$$mGA{P}_{ss}\,=\,\frac{{k}_{on}^{mGAP}}{{k}_{off}^{mGAP}}+\frac{{k}_{feedback}}{{k}_{off}^{mGAP}}{f}_{act}(tGE{F}_{ss})\cdot {f}_{act}(t{G}_{ss}^{* })$$

for the AND logic gate, and

$$mGA{P}_{ss}\,=\,\frac{{k}_{on}^{mGAP}}{{k}_{off}^{mGAP}}+\frac{{k}_{feedback}}{{k}_{off}^{mGAP}}\left({f}_{act}(tGE{F}_{ss})+{f}_{act}(t{G}_{ss}^{* })\right)$$

for the OR logic gate. By substituting the *mGAP*_*ss*_ with the above equations, we got the $m{G}_{ss}^{* }$ as follows:

$$m{G}_{ss}^{* }\,=\,\frac{{F}_{1}({R}_{ss}^{* })}{{F}_{1}({R}_{ss}^{* })+{h}_{Hill}(m{G}_{ss}^{* },{k}_{feedback})}$$

where ${F}_{1}({R}_{ss}^{* })$ has been defined in the previous section. The ${h}_{Hill}(m{G}_{ss}^{* },{k}_{feedback})$ is defined as:

$${h}_{Hill}(m{G}_{ss}^{* },{k}_{feedback})=\left\{\begin{array}{l}\frac{{k}_{on}^{mG}}{{k}_{off}^{mG}}{f}_{act}\left(\frac{{k}_{on}^{mGAP}}{{k}_{off}^{mGAP}}+\frac{{k}_{feedback}}{{k}_{off}^{mGAP}}{f}_{act}\left(\frac{{k}_{on}^{tGEF}}{{k}_{off}^{tGEF}}{f}_{act}(m{G}_{ss}^{* })\right)\cdot {f}_{act}\left(\frac{{f}_{act}(\frac{{k}_{on}^{tGEF}}{{k}_{off}^{tGEF}}{f}_{act}(m{G}_{ss}^{* }))}{{f}_{act}(\frac{{k}_{on}^{tGEF}}{{k}_{off}^{tGEF}}{f}_{act}(m{G}_{ss}^{* }))+{b}_{H}}\right)\right)\quad \\ \,{{\mbox{for the AND logic gate,}}}\,\quad \\ \frac{{k}_{on}^{mG}}{{k}_{off}^{mG}}{f}_{act}\left(\frac{{k}_{on}^{mGAP}}{{k}_{off}^{mGAP}}+\frac{{k}_{feedback}}{{k}_{off}^{mGAP}}\left({f}_{act}\left(\frac{{k}_{on}^{tGEF}}{{k}_{off}^{tGEF}}{f}_{act}(m{G}_{ss}^{* })\right)+{f}_{act}\left(\frac{{f}_{act}(\frac{{k}_{on}^{tGEF}}{{k}_{off}^{tGEF}}{f}_{act}(m{G}_{ss}^{* }))}{{f}_{act}(\frac{{k}_{on}^{tGEF}}{{k}_{off}^{tGEF}}{f}_{act}(m{G}_{ss}^{* }))+{b}_{H}}\right)\right)\right)\quad \\ \,{{\mbox{for the OR logic gate.}}}\,\quad \end{array}\right.$$

For simplicity, we still used *x* and *y* to denote the ${R}_{ss}^{* }$ and $m{G}_{ss}^{* }$, respectively. Then relation between ${R}_{ss}^{* }$ and $m{G}_{ss}^{* }$ can be rewritten as:

$$y=\frac{{F}_{1}(x)}{{F}_{1}(x)+{h}_{Hill}(y,{k}_{feedback})}.$$

As we have shown in the previous section, the sign of the $\frac{\partial (y/{y}_{max})}{\partial {k}_{feedback}}$ is determined by $\frac{1}{y}\frac{\partial y}{\partial {k}_{feedback}}-\frac{1}{{y}_{max}}\frac{\partial {y}_{max}}{\partial {k}_{feedback}}$. The expression of $\frac{1}{y}\frac{\partial y}{\partial {k}_{feedback}}$ is shown as follows:

$$\begin{array}{ll}\frac{1}{y}\frac{\partial y}{\partial {k}_{feedback}}\,=\,-\,\frac{1}{y}\frac{{F}_{1}(x)\frac{\partial {h}_{Hill}}{\partial {k}_{feedback}}}{{F}_{1}(x)\frac{\partial {h}_{Hill}}{\partial y}+{[{F}_{1}(x)+{h}_{Hill}(y,{k}_{feedback})]}^{2}}\\ \qquad\qquad\,=\,-\,\frac{1}{y}\frac{1}{\frac{\partial {h}_{Hill}}{\partial y}\frac{1}{\frac{\partial {h}_{Hill}}{\partial {k}_{feedback}}}+{[{F}_{1}(x)+{h}_{Hill}(y,{k}_{feedback})]}^{2}\frac{1}{{F}_{1}(x)\frac{\partial {h}_{Hill}}{\partial {k}_{feedback}}}}\\ \qquad\qquad\,=\,-\,\frac{1}{\frac{\partial {h}_{Hill}}{\partial y}\frac{y}{\frac{\partial {h}_{Hill}}{\partial {k}_{feedback}}}+[{F}_{1}(x)+{h}_{Hill}(y,{k}_{feedback})]\frac{1}{\frac{\partial {h}_{Hill}}{\partial {k}_{feedback}}}}\\ \qquad\qquad\,=\,-\,\frac{1}{\frac{\partial {h}_{Hill}}{\partial y}\frac{y}{\frac{\partial {h}_{Hill}}{\partial {k}_{feedback}}}+\frac{1}{1-y}\frac{{h}_{Hill}(y,{k}_{feedback})}{\frac{\partial {h}_{Hill}}{\partial {k}_{feedback}}}}.\end{array}$$

To further simplify this equation, we rewrote the *h*_*Hill*_(*y*) as *G*(*z*), where $G(z)=\frac{{k}_{on}^{mG}}{{k}_{off}^{mG}}{f}_{act}(z)$, $z=\frac{{k}_{on}^{mGAP}}{{k}_{off}^{mGAP}}+{k}_{feedback}\theta (y)$, $\theta (y)=\frac{1}{{k}_{off}^{mGAP}}\left({f}_{act}\left(\frac{{k}_{on}^{tGEF}}{{k}_{off}^{tGEF}}{f}_{act}(y)\right){f}_{act}\left(\frac{{f}_{act}\left(\frac{{k}_{on}^{tGEF}}{{k}_{off}^{tGEF}}{f}_{act}(y)\right)}{{f}_{act}\left(\frac{{k}_{on}^{tGEF}}{{k}_{off}^{tGEF}}{f}_{act}(y)\right)+{b}_{H}}\right)\right)$ for the AND logic gate and $\frac{1}{{k}_{off}^{mGAP}}\left({f}_{act}\left(\frac{{k}_{on}^{tGEF}}{{k}_{off}^{tGEF}}{f}_{act}(y)\right)+{f}_{act}\left(\frac{{f}_{act}\left(\frac{{k}_{on}^{tGEF}}{{k}_{off}^{tGEF}}{f}_{act}(y)\right)}{{f}_{act}\left(\frac{{k}_{on}^{tGEF}}{{k}_{off}^{tGEF}}{f}_{act}(y)\right)+{b}_{H}}\right)\right)$ for the OR logic gate. Therefore, the derivative of the second term in the denominator with respect to the *y* is given by:

$$\begin{array}{ll}\frac{\partial }{\partial y}\left(\frac{1}{1-y}\frac{{h}_{Hill}}{\frac{\partial {h}_{Hill}}{\partial {k}_{feedback}}}\right)\\ \,=\,\frac{\partial }{\partial y}\left(\frac{1}{1-y}\frac{{h}_{Hill}}{{G}^{{\prime} }(z)\theta (y)}\right)\\ \,=\,\frac{1}{{(1-y)}^{2}}\left[\frac{{h}_{Hill}}{{G}^{{\prime} }(z)\theta (y)}+(1-y)\frac{\frac{\partial {h}_{Hill}}{\partial y}{G}^{{\prime} }(z)\theta (y)-{h}_{Hill}\left({k}_{feedback}{G}^{{\prime\prime} }(z){\theta }^{{\prime} }(y)\theta (y)+{G}^{{\prime} }(z){\theta }^{{\prime} }(y)\right)}{{({G}^{{\prime} }(z)\theta (y))}^{2}}\right].\end{array}$$

When *y* goes to 1, this equation is larger than 0, because the first term is much larger than the second term and the first term is always positive. When *y* is 0, *θ*(0) = 0, and $\frac{\partial }{\partial y}(\frac{1}{1-y}\frac{{h}_{Hill}}{\frac{\partial {h}_{Hill}}{\partial {k}_{feedback}}})=\frac{{h}_{Hill}}{{G}^{{\prime} }(z)\theta (y)}+\frac{\frac{\partial {h}_{Hill}}{\partial y}}{{G}^{{\prime} }(z)\theta (y)}-\frac{{h}_{Hill}{G}^{{\prime} }(z){\theta }^{{\prime} }(y)}{{({G}^{{\prime} }(z)\theta (y))}^{2}}$; due to $\frac{1}{\theta {(y)}^{2}}\gg \frac{1}{\theta (y)}$ when *y* goes to 0, the third term dominates and thus $\frac{\partial }{\partial y}(\frac{1}{1-y}\frac{{h}_{Hill}}{\frac{\partial {h}_{Hill}}{\partial {k}_{feedback}}})$ is smaller than 0. To now, we have proved that the second term in the denominator of the function $\frac{1}{y}\frac{\partial y}{\partial {k}_{feedback}}$ is decreasing for the small *y* and increasing for enough large *y*. If we neglected the effect of the first term, we can conclude that $\frac{1}{y}\frac{\partial y}{\partial {k}_{feedback}}\ge \frac{1}{{y}_{max}}\frac{\partial {y}_{max}}{\partial {k}_{feedback}}$ for the small *y*_*max*_ and $\frac{1}{y}\frac{\partial y}{\partial {k}_{feedback}}\le \frac{1}{{y}_{max}}\frac{\partial {y}_{max}}{\partial {k}_{feedback}}$ for the large *y*_*max*_. Since the large *y*_*max*_ usually corresponds to the case that the normalized mGTPase curve is higher than the receptor curve, the negative sign of the $\frac{\partial (y/{y}_{max})}{\partial {k}_{feedback}}$ indicates the closer distance between two curves with increased feedback. Similarly, the small *y*_*max*_ also suggests the closer distance between two curves with increased feedback, because at that time the normalized mGTPase curve is usually lower than the receptor curve. To now, we have finished the proof of Fig. 3c. The DoRA of tGTPase follows the same rule, and it can be proven using the same method shown in the previous section.

### Analysis for different logic gate

#### The mass action model

In the mass action model, the OR logic gates show a little better DoRA behavior than AND logic gate. We used ${k}_{feedback}^{AND}$ and ${k}_{feedback}^{OR}$ to represent the negative feedback strength for the system with the AND logic gate and the system with the OR logic gate, respectively. The requirement about the same mG^*^ activation level (i.e., $m{G}_{ss,max}^{* }$) indicates the same mGAP level when the stimulus *S* → *∞*, because the mGEF is not affected by logic gates. Since the mGAP level is the same, the AND and OR logic gates show the same regulatory strength to the mGAP, leading to the following equation:

$${k}_{feedback}^{AND}\underbrace{{h}_{1}(m{G}_{ss,max}^{* })}_{\begin{array}{c}tGE{F}_{ss,max}\end{array}}\underbrace{{h}_{2}(m{G}_{ss,max}^{* })}_{\begin{array}{c}t{G}_{ss,max}^{* }\end{array}}={k}_{feedback}^{OR}(\underbrace{{h}_{1}(m{G}_{ss,max}^{* })}_{\begin{array}{c}tGE{F}_{ss,max}\end{array}}+\underbrace{{h}_{2}(m{G}_{ss,max}^{* })}_{\begin{array}{c}t{G}_{ss,max}^{* }\end{array}})$$

where ${h}_{1}(m{G}_{ss}^{* })$ and ${h}_{2}(m{G}_{ss}^{* })$ denote *tGEF*_*ss*_ and $t{G}_{ss}^{* }$, respectively. All variables are steady-state values when the stimulus *S* → *∞*. Because the function $\frac{{k}_{feedback}^{OR}}{{k}_{feedback}^{AND}}\left(\frac{1}{{h}_{1}(x)}+\frac{1}{{h}_{2}(x)}\right)$ is a decreasing function of *x*, we had

22 $$\frac{{k}_{feedback}^{OR}}{{k}_{feedback}^{AND}}\left(\frac{1}{{h}_{1}(m{G}_{ss}^{* })}+\frac{1}{{h}_{2}(m{G}_{ss}^{* })}\right)\ge \frac{{k}_{feedback}^{OR}}{{k}_{feedback}^{AND}}\left(\frac{1}{{h}_{1}(m{G}_{ss,max}^{* })}+\frac{1}{{h}_{2}(m{G}_{ss,max}^{* })}\right)=1$$

The left-hand side can be rewritten as $\frac{{k}_{feedback}^{OR}({h}_{1}(m{G}_{ss}^{* })+{h}_{2}(m{G}_{ss}^{* }))}{{k}_{feedback}^{AND}{h}_{1}(m{G}_{ss}^{* }){h}_{2}(m{G}_{ss}^{* })}$. So, ${k}_{feedback}^{OR}({h}_{1}(m{G}_{ss}^{* })+{h}_{2}(m{G}_{ss}^{* }))$ is larger than ${k}_{feedback}^{AND}{h}_{1}(m{G}_{ss}^{* }){h}_{2}(m{G}_{ss}^{* })$; this indicates that the system with the OR logic gate has stronger feedback strength and thus lower mG^*^ level when compared to the system with the AND logic gate. Combined with the fact that $m{G}_{ss}^{* }/m{G}_{ss,max}^{* }$ curve is always higher than the receptor curve in the mass action model, the distance between these two curves in the system with the OR logic gate is smaller than that with AND logic gate (Fig. 4b). This implies the better DoRA performance of the OR logic in the mass action model. However, this advantage is small (See main texts for more explanations).

### Reporting summary

Further information on research design is available in the Nature Research Reporting Summary linked to this article.

## Supplementary information

Reporting Summary

Supplementary Information

## Data Availability

The Matlab data sets generated by this work are available on the github (https://github.com/RangamaniLabUCSD/Qiao_et_al_Dose-response-alignment).

## Code availability

All numerical simulations were done in Matlab, and the Matlab code is available on the github (https://github.com/RangamaniLabUCSD/Qiao_et_al_Dose-response-alignment).
