# Supplementary material for: Design principles of improving the dose-response alignment in coupled GTPase switches
Source: NPJ Syst Biol Appl. 2023 Jan 31;9:3. doi: 10.1038/s41540-023-00266-9 (PMC9889403; doi:10.1038/s41540-023-00266-9)
Supplement: Supplementary file 2 — Supplementary Information [file 41540_2023_266_MOESM2_ESM.pdf]

Supplementary information for "Design principles of improving the dose-response alignment in coupled GTPase switches"

This file contains:

Supplementary Table 1

Supplementary Figure 1

Supplementary Figure 2

Supplementary Figure 3

Supplementary Figure 4

Supplementary Figure 5

Supplementary Figure 6

Supplementary Figure 7

Supplementary Figure 8

Supplementary Figure 9

Supplementary Table 1: Parameters used in numerical simulations

Stimulus S	10^x, where x is uniformly distributed in [-5, 3] with the increment 0.2
k_{on}^i, $i = mGEF, tGEF$	1
k_{off}^i, $i = R^*, mGEF,$ $mG^*, tGEF, tG^*, mGAP$	1
$tGAP$	0.5
K	0.5
n (unless otherwise specified)	1.5
**Parameters used in Figure 2**	
$k_{feedback}$	0
k_{on}^{mGAP}	[0.01:0.02:0.1, 0.15:0.05:0.4, 0.5:0.1:1]
$(k_{on}^R, k_{on}^{mG}, k_{on}^{tG})$ in the upper panel in b	$(0.5, 10^0, 10^0)$
$k_{on}^R, k_{on}^{mG}, k_{on}^{tG}$ in the middle and lower panels in b	k_{on}^R=0.5. k_{on}^{mG} and k_{on}^{tG} are 10^x, where x is uniformly distributed in [-2, 2] with the increment 0.2 in the lower panel or 0.1 in the middle panel
$(k_{on}^R, k_{on}^{mG}, k_{on}^{tG})$ (orange) in the upper panel in d	$(10^{0.2}, 10^{0.2}, 1)$
$(k_{on}^R, k_{on}^{mG}, k_{on}^{tG})$ (magenta) in the upper panel in d	$(10^{-0.8}, 10^0, 1)$
$(k_{on}^R, k_{on}^{mG}, k_{on}^{tG})$ (purple) in the upper panel in d	$(10^{-1.2}, 10^{-1.2}, 1)$
$k_{on}^R, k_{on}^{mG}, k_{on}^{tG}$ in the middle and lower panels in d	k_{on}^R and k_{on}^{mG} are 10^x, where x is uniformly distributed in [-2, 2] with the increment 0.2. $k_{on}^{tG} = 0.01, 1,$ or 10
**Parameters used in Figure 3**	
k_{on}^{mGAP}	0.5
$k_{feedback}$	0 and 10^x, x is uniformly distributed in [-1, 2.6] with the increment 0.2
$(k_{on}^R, k_{on}^{mG}, k_{on}^{tG})$ for the example 1 in a	$(1, 10, 10)$
$(k_{on}^R, k_{on}^{mG}, k_{on}^{tG})$ for the example 2 in a	$(0.1, 10^{-0.5}, 10^{-0.5})$
$(k_{on}^R, k_{on}^{mG}, k_{on}^{tG})$ (purple) in the upper two panels in b	$(0.5, 10^{-1.4}, 10^1)$

$(k_{on}^{R}, k_{on}^{mG}, k_{on}^{tG})$ (orange) in the upper left panel in b	$(0.5, 10^{1.4}, 10^{2})$
$(k_{on}^{R}, k_{on}^{mG}, k_{on}^{tG})$ (magenta) in the upper left panel in b	$(0.5, 10^{0.4}, 10^{1})$
$(k_{on}^{R}, k_{on}^{mG}, k_{on}^{tG})$ (black) in the upper right panel in b	$(0.5, 10^{-0.6}, 10^{0.6})$
$(k_{on}^{R}, k_{on}^{mG}, k_{on}^{tG})$ (orange) in the upper right panel in b	$(0.5, 10^{1}, 10^{1})$
$k_{on}^{R}, k_{on}^{mG}, k_{on}^{tG}$ in the middle and lower panels in b	Same as those in Figure 2b
$(k_{on}^{R}, k_{on}^{mG}, k_{on}^{tG})$ for the example 1 in c	$(10^{0.4}, 10^{0.4}, 1)$
$(k_{on}^{R}, k_{on}^{mG}, k_{on}^{tG})$ for the example 2 in c	$(10^{-0.6}, 10^{-0.6}, 1)$
$(k_{on}^{R}, k_{on}^{mG}, k_{on}^{tG})$ (orange in right upper corner) in upper panels in d	$(10^{0.4}, 10^{0.4}, 1)$
$(k_{on}^{R}, k_{on}^{mG}, k_{on}^{tG})$ (magenta) in upper panels in d	$(10^{-1}, 10^{1}, 1)$
$(k_{on}^{R}, k_{on}^{mG}, k_{on}^{tG})$ (orange in left bottom corner) in upper panels in d	$(10^{-0.6}, 10^{-0.6}, 1)$
$(k_{on}^{R}, k_{on}^{mG}, k_{on}^{tG})$ (black) in upper panels in d	$(10^{1}, 10^{-0.6}, 1)$
$k_{on}^{R}, k_{on}^{mG}, k_{on}^{tG}$ in the middle and lower panels in d	Same as those in Figure 2d
**Parameters used in Supplementary Figure 4 and Supplementary Figure 6**	
k_{on}^{mGAP}	0.5
$(k_{on}^{R}, k_{on}^{mG}, k_{on}^{tG})$	(1, 10, 10)
$k_{feedback}$	1, 5, or 10
**Parameters used in Supplementary Figure 5 and Supplementary Figure 7**	
k_{on}^{mGAP}	0.5
$(k_{on}^{R}, k_{on}^{mG}, k_{on}^{tG})$	$(0.1, 10^{-0.5}, 10^{-0.5})$
$k_{feedback}$	1, 5, or 10

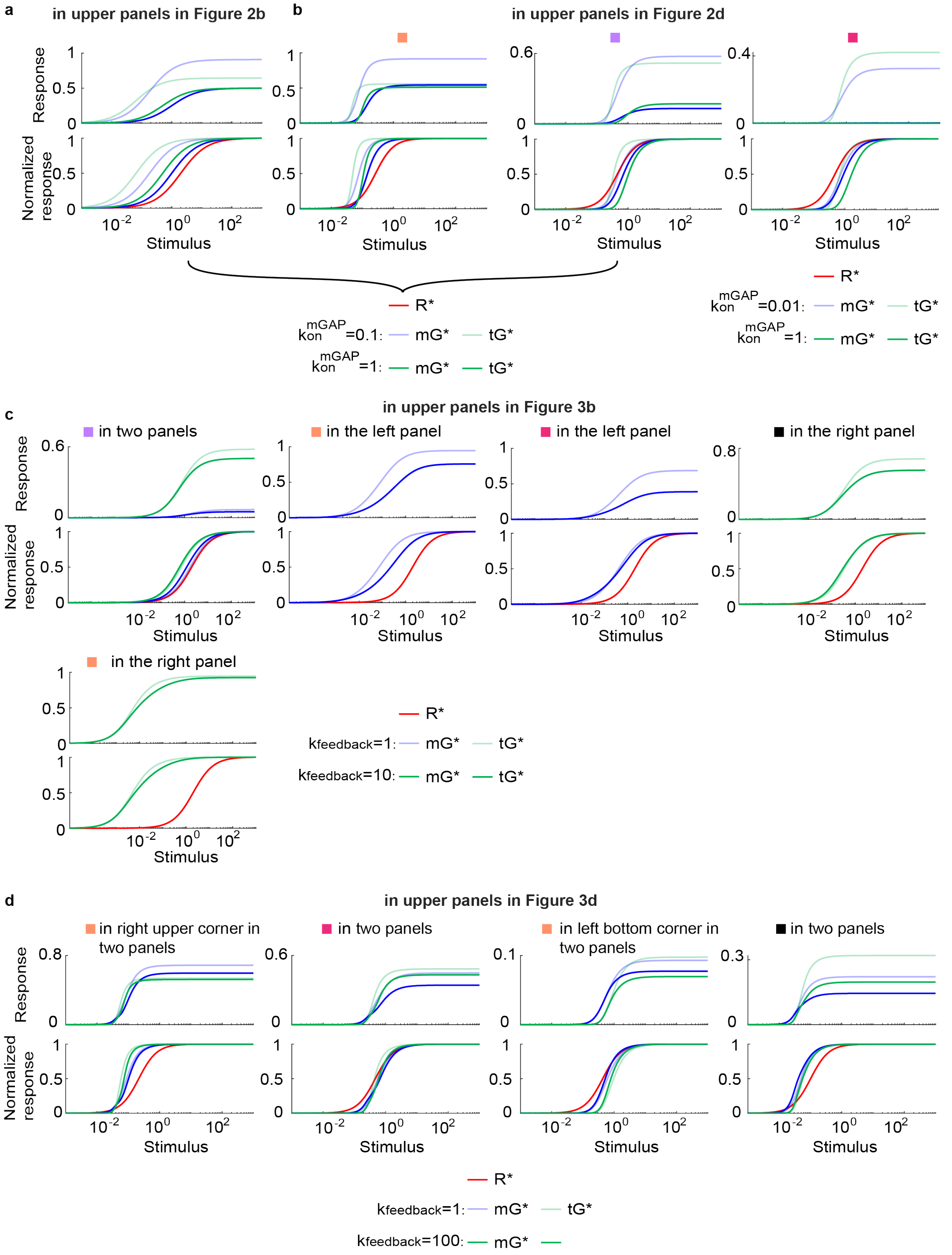

Supplementary Figure 1: The dose-response curves for the parameter sets in Supplementary Table 1. (a-d) Dose-response curve corresponding the kinetic parameter set used in upper panels in Figure 2b, 2d, 3b, and 3d.

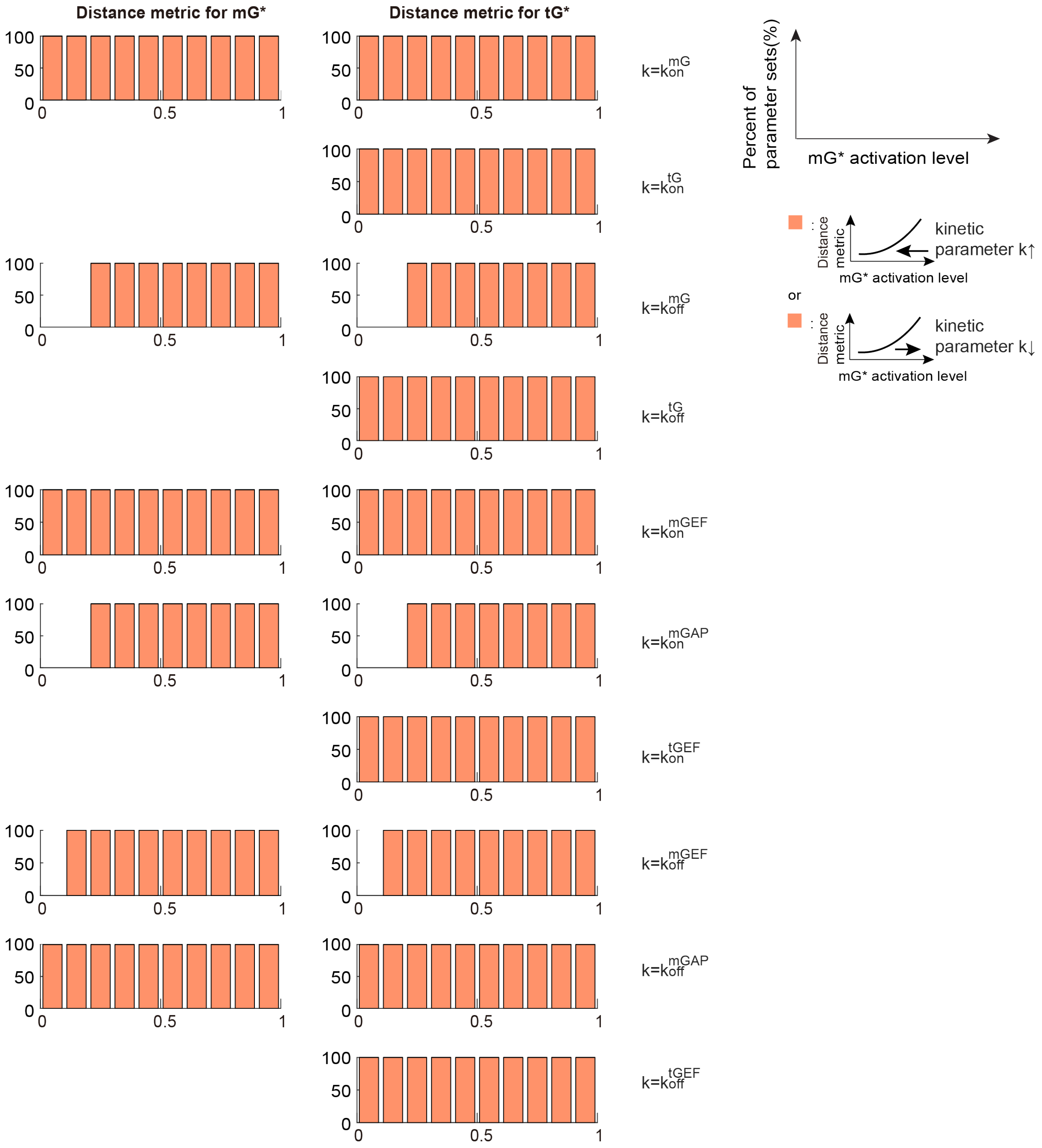

Supplementary Figure 2: The distributions of trends for every kinetic parameter in coupled switches without feedback when using the mass action model. Same plots as in the middle panel Figure 2b except that the varied kinetic parameter is different. The varied kinetic parameter is shown on the right. Some of the left panels are missing, because this kinetic parameter cannot affect the distance metric for mGTPase. One thousand parameter sets are randomly assigned in a logarithmic scale in the whole parameter space using Latin hypercubic sampling, and each parameter is in the range $[10^{-2}, 10^{1}]$. Then the varied parameter is uniformly changed from 10^{-2} to 10^{1} in a logarithmic scale.

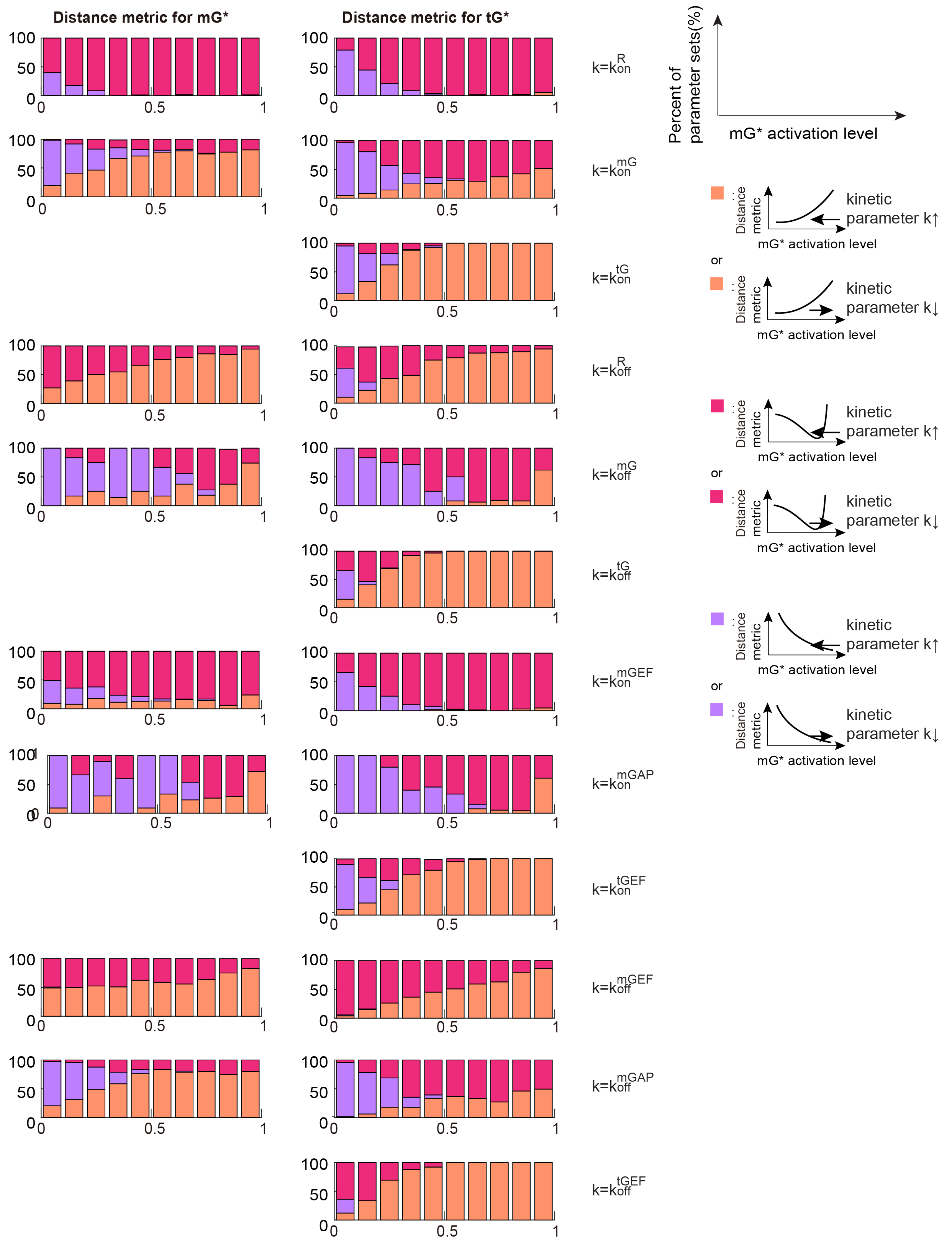

Supplementary Figure 3: Same plot as Supplementary Figure 2 except that the Hill-function kinetics is adopted.

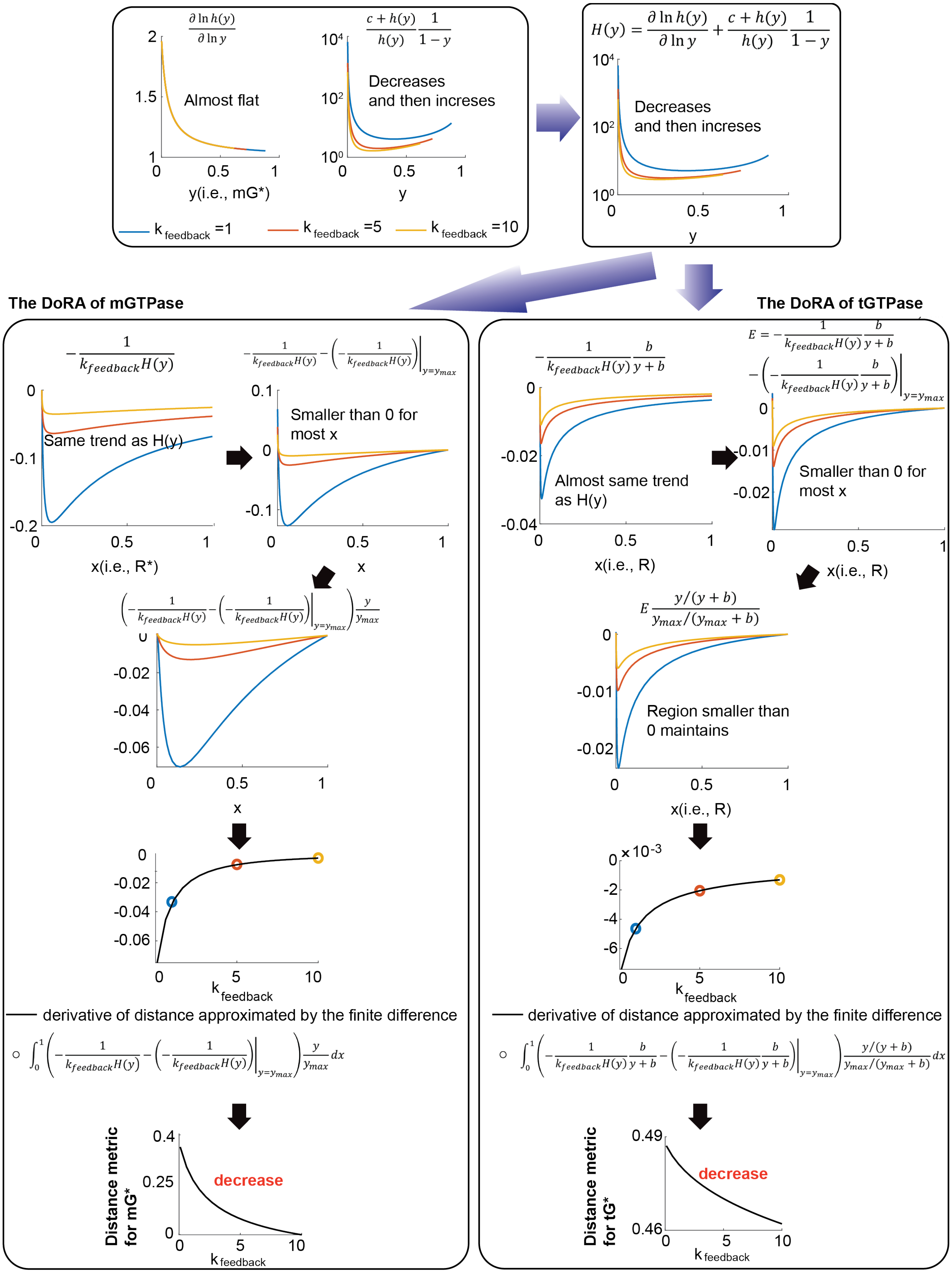

Supplementary Figure 4: DoRA of the m- and tGTPase is improved with increased feedback strength in the mass action model. Here AND gate is applied. See Supplementary Table 1 for parameters.

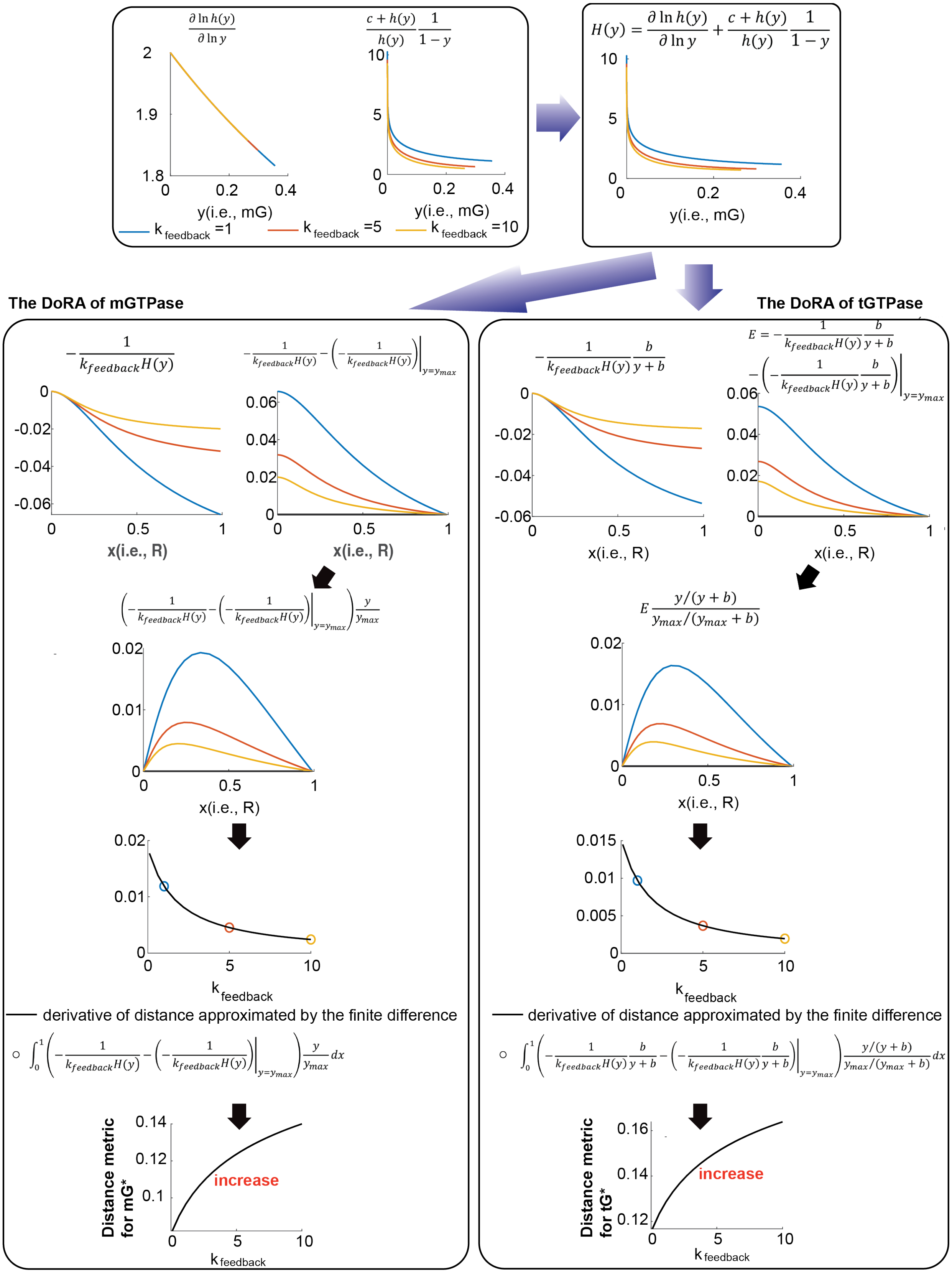

Supplementary Figure 5: For some kinetic parameter sets, DoRA is impaired by increased feedback strength in the mass action model. Here AND gate is applied. See Supplementary Table 1 for parameters.

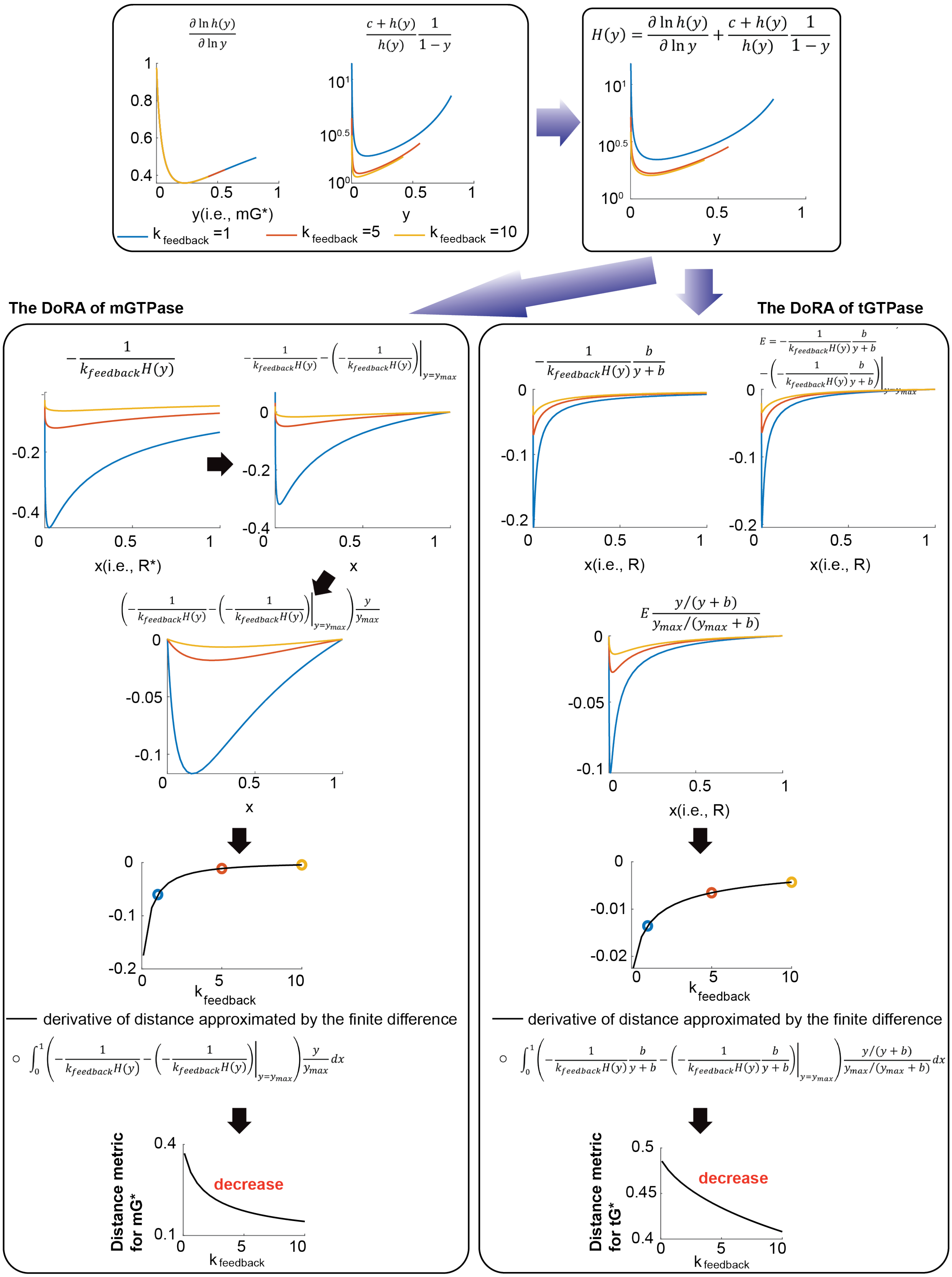

Supplementary Figure 6: Same plot as Supplementary Figure 4 but OR logic is used to model the negative feedback. See Supplementary Table 1 for parameters.

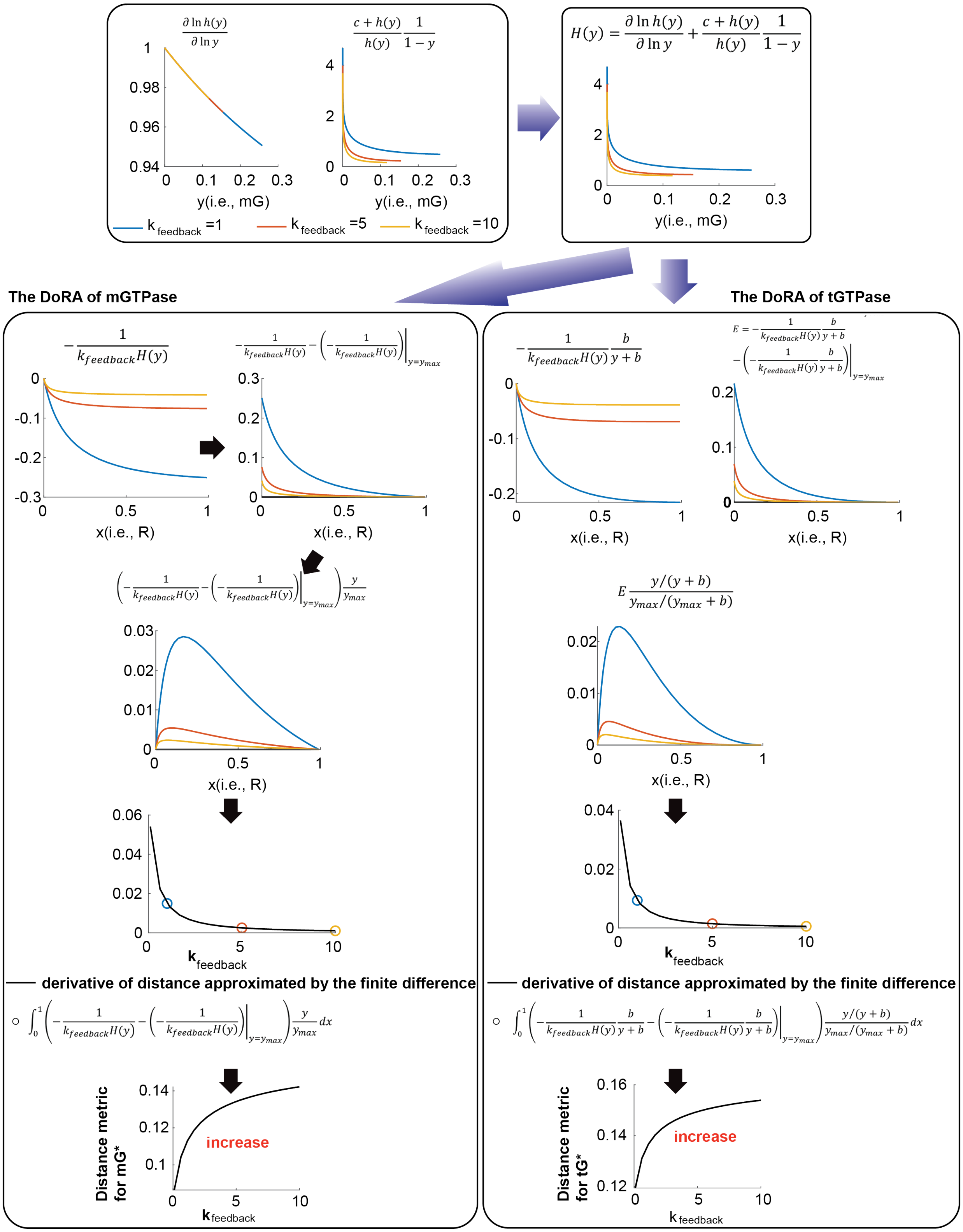

Supplementary Figure 7: Same plot as Supplementary Figure 5 but OR logic is used to model the negative feedback. See Supplementary Table 1 for parameters.

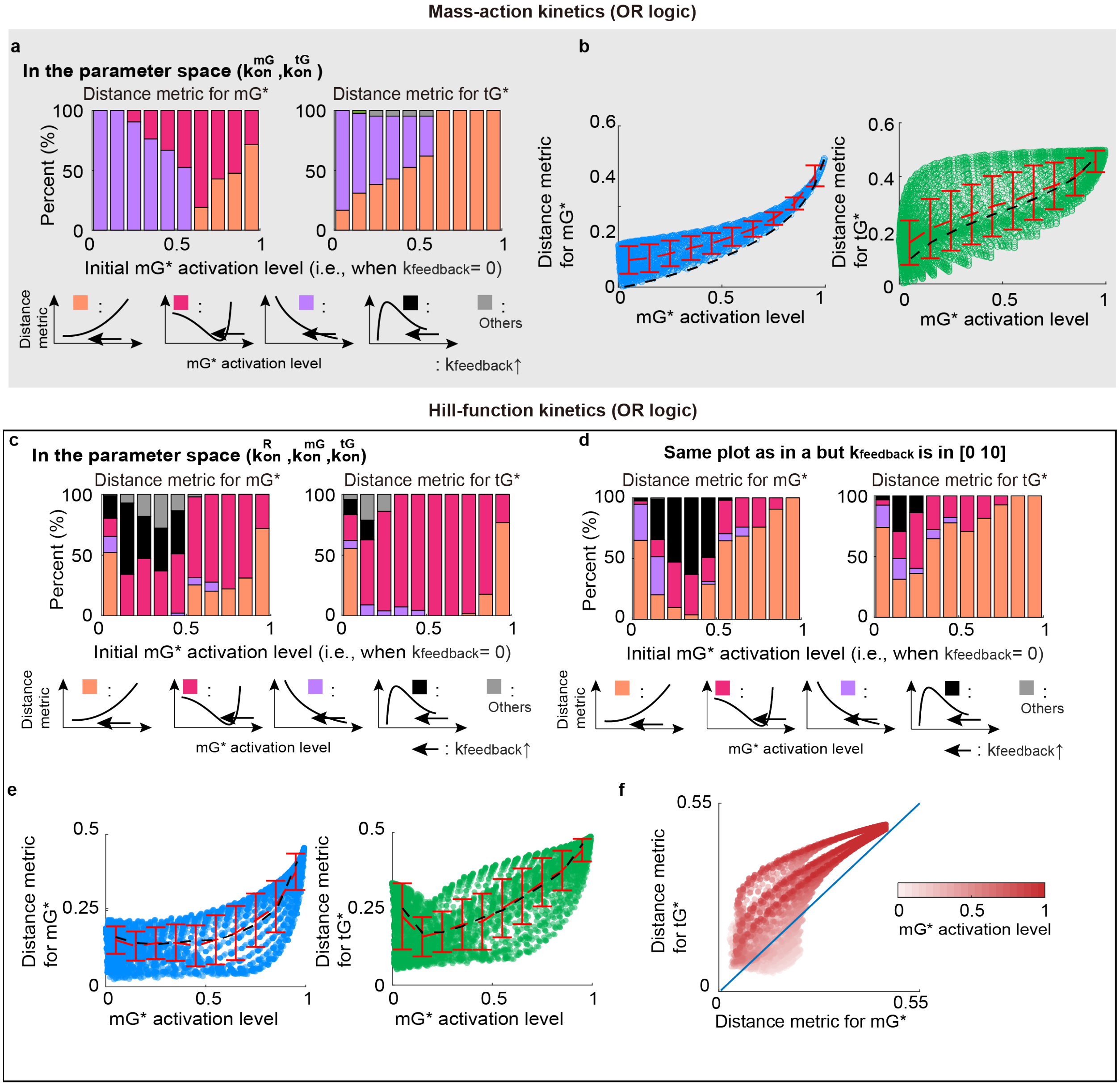

Supplementary Figure 8: The simulation results when using OR logic gate. (a-b) Same plot as the middle and bottom panels in Figure 3b except that OR logic is used to model the negative feedback. (c-e) Same plot as the middle and bottom panels in Figure 3d except the logic gate. The panel c and d has different ranges of $k_{feedback}$. (f) Same plot as the right panel in Figure 5d except the logic gate.

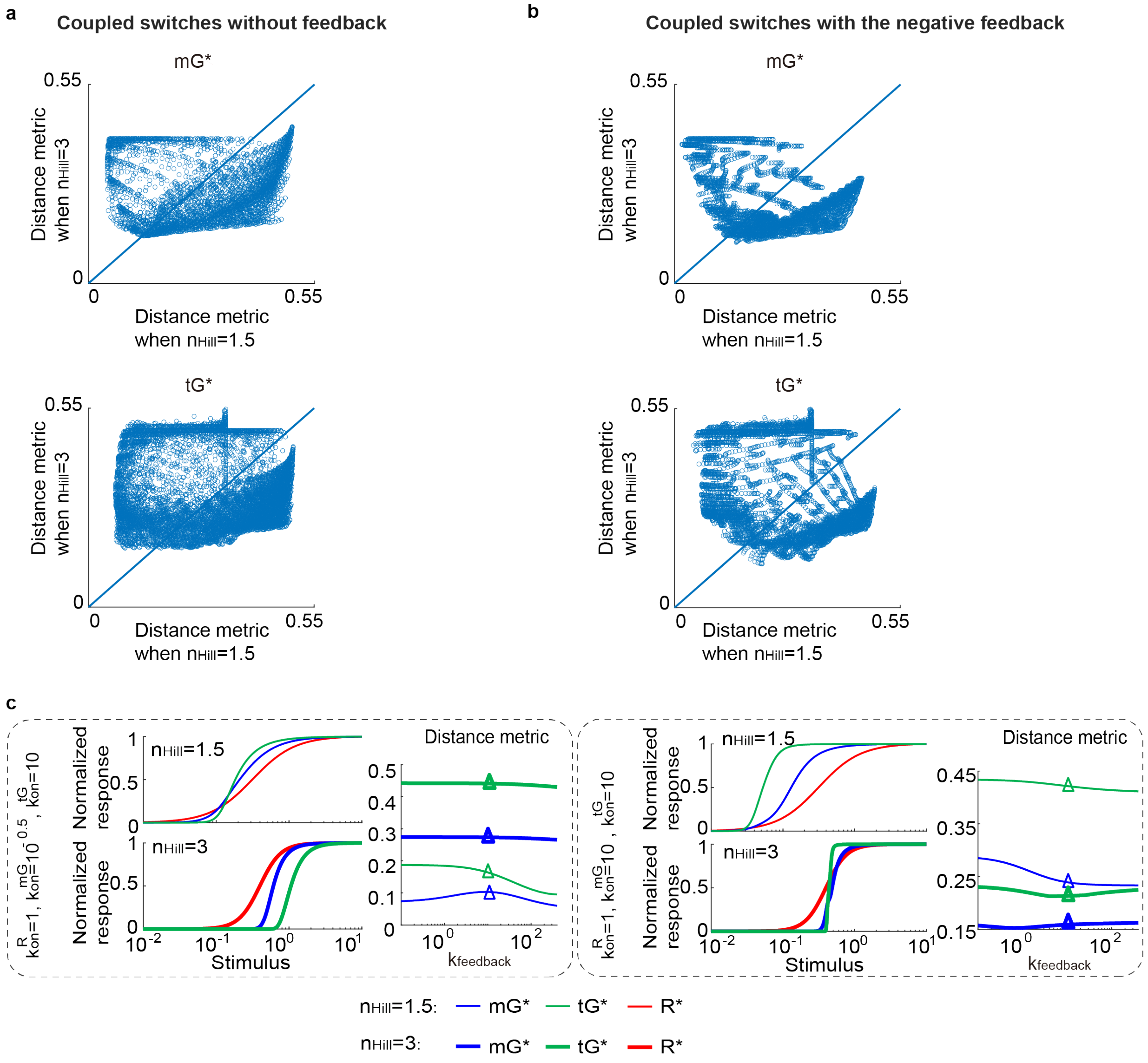

Supplementary Figure 9: The effect of the Hill coefficient. (a-b) The scatter plots of the distance metric with n_{Hill} 1.5 versus that with n_{Hill} 3 in the parameter space $\{k_{on}^{R}, k_{on}^{mG}, k_{on}^{tG}\}$ for the circuit without (a) or with feedback (b). The n_{Hill} is the Hill coefficient n mentioned in the first section. (c) Two examples showing the different effects of n_{Hill} on DoRA.
